# Lignin deconstruction by anaerobic fungi

**DOI:** 10.1038/s41564-023-01336-8

**Published:** 2023-03-09

**Authors:** Thomas S. Lankiewicz, Hemant Choudhary, Yu Gao, Bashar Amer, Stephen P. Lillington, Patrick A. Leggieri, Jennifer L. Brown, Candice L. Swift, Anna Lipzen, Hyunsoo Na, Mojgan Amirebrahimi, Michael K. Theodorou, Edward E. K. Baidoo, Kerrie Barry, Igor V. Grigoriev, Vitaliy I. Timokhin, John Gladden, Seema Singh, Jenny C. Mortimer, John Ralph, Blake A. Simmons, Steven W. Singer, Michelle A. O’Malley

**Affiliations:** 1 Department of Chemical Engineering, University of California Santa Barbara, Santa Barbara, CA USA; 2 Department of Ecology, Evolution, and Marine Biology, University of California Santa Barbara, Santa Barbara, CA USA; 3 Joint BioEnergy Institute, Emeryville, CA USA; 4 Department of Biomaterials and Biomanufacturing, Sandia National Laboratories, Livermore, CA USA; 5 Environmental Genomics and Systems Biology Division, Lawrence Berkeley National Laboratory, Berkeley, CA USA; 6 Department of Environmental Health Sciences, University of South Carolina, Columbia, SC USA; 7 Department of Energy Joint Genome Institute, Lawrence Berkeley National Laboratory, Berkeley, CA USA; 8 Department of Agriculture and Environment, Harper Adams University, Newport, UK; 9 Biological Systems and Engineering Division, Lawrence Berkeley National Laboratory, Berkeley, CA USA; 10 Department of Plant and Microbial Biology, University of California Berkeley, Berkeley, CA USA; 11 Great Lakes Bioenergy Research Center, Madison, WI USA; 12 School of Agriculture, Food and Wine, Waite Research Institute, University of Adelaide, Glen Osmond, South Australia Australia; 13 Department of Biochemistry, University of Wisconsin Madison, Madison, WI USA

**Keywords:** Applied microbiology, Biocatalysis, Fungi

## Abstract

Lignocellulose forms plant cell walls, and its three constituent polymers, cellulose, hemicellulose and lignin, represent the largest renewable organic carbon pool in the terrestrial biosphere. Insights into biological lignocellulose deconstruction inform understandings of global carbon sequestration dynamics and provide inspiration for biotechnologies seeking to address the current climate crisis by producing renewable chemicals from plant biomass. Organisms in diverse environments disassemble lignocellulose, and carbohydrate degradation processes are well defined, but biological lignin deconstruction is described only in aerobic systems. It is currently unclear whether anaerobic lignin deconstruction is impossible because of biochemical constraints or, alternatively, has not yet been measured. We applied whole cell-wall nuclear magnetic resonance, gel-permeation chromatography and transcriptome sequencing to interrogate the apparent paradox that anaerobic fungi (Neocallimastigomycetes), well-documented lignocellulose degradation specialists, are unable to modify lignin. We find that Neocallimastigomycetes anaerobically break chemical bonds in grass and hardwood lignins, and we further associate upregulated gene products with the observed lignocellulose deconstruction. These findings alter perceptions of lignin deconstruction by anaerobes and provide opportunities to advance decarbonization biotechnologies that depend on depolymerizing lignocellulose.

## Abstract (web summary)

Fungi from the Neocallimastigomycetes taxonomic class break bonds in lignin during the anaerobic deconstruction of whole plant cell walls. This finding challenges the paradigm that only certain aerobic organisms break down lignin.

## Main

Lignin is an irregular phenylpropanoid biopolymer and one of the three major components of lignocellulose, the composite material forming secondary cell walls in higher plants. Lignin can comprise up to one-third of the dry mass of plant cell walls, making it the second most abundant biopolymer in the terrestrial biosphere, after cellulose, and the most abundant aromatic polymer^1,2^. The aromatic groups in lignin impart essential properties to plant cell walls, including resistance to degradation, structural rigidity and hydrophobicity, properties that facilitate fluid transport, defence against pathogens and biomass accumulation^3^. Plant cells synthesize lignin from three different primary *p*-hydroxycinnamyl alcohols: *p-*coumaryl, coniferyl and sinapyl alcohol. These monomers polymerize through free-radical coupling mechanisms, giving rise to *p*-hydroxyphenyl (H), guaiacyl (G) and syringyl (S) subunits that are present in varying ratios in different lignins^2^. Stochastic polymerization processes combined with variations in S:G:H proportions contribute to heterogeneity, diverse bond types and varying degrees of branching in lignin structures^2^. Lignin’s high recalcitrance defines its biogeochemical role as a carbon sink and presents a significant challenge for biotechnologies seeking to sustainably produce commodity chemicals from lignocellulose^4–7^.

Current descriptions of biological depolymerization and modification of lignin focus on aerobic systems, and they are primarily associated with the fungal sub-kingdom of Dikarya^6,8^. Although some members of Dikarya, such as ascomycete yeasts, are facultative anaerobes, lignin-degrading organisms, such as white rots, thrive in the presence of molecular oxygen^6,9^. Characterized lignin-modifying enzymes are limited in diversity and reflect the aerobic nature of their hosts; most rely on oxygen-dependent mechanisms that are probably unavailable under anaerobic conditions^6,9^. Many of these mechanisms indirectly (non-enzymatically) depolymerize lignin through the generation of organic free radicals and are therefore designated as lignin-active enzymes rather than lignin enzymes^6,9^. The oxygen dependence of characterized lignin-active enzymes has led to the widely accepted view that biological lignin deconstruction cannot occur in anaerobic environments^6^. The described lignin-active enzymes are categorized as laccases, lignin peroxidases, manganese peroxidases, versatile peroxidases, dye-decolourizing peroxidases, other oxidases and β-etherases^6,9^. Aerobic bacteria also produce a subset of these enzymes, but no anaerobic organism, bacterial or otherwise, possesses any known lignin-active enzyme^9,10^.

Anaerobic microbial communities, such as herbivore gut microbiomes, rapidly process lignocellulose under anaerobic conditions, producing greenhouse gases on scales that directly impact the global climate^11,12^. However, the fate of lignin in anaerobic environments remains largely unknown^1,13^. At a minimum, herbivore gut communities, and other microbes decomposing lignocellulose anaerobically, must displace lignin from plant cell walls to access cellulose and hemicelluloses, the primary carbon sources for microbiome and animal metabolism. Some previous studies have collected indirect evidence of lignin modification by anaerobic bacteria, but these studies are unable to establish the natural occurrence of anaerobic lignin deconstruction because they interrogate changes to Kraft lignin, a lignin-derived extract that has already been modified from naturally occurring lignins^14–19^. Insights into whether and, if so, how anaerobic microbes remove native lignins to acquire carbohydrates will inform geochemical models by helping to define carbon remineralization processes in diverse environments in which lignocellulose is anaerobically deconstructed^13^.

Organisms from Neocallimastigomycetes are early-branching anaerobic fungi broadly distributed in herbivore digestive tracts that excel at degrading lignocellulose and occupy a keystone role in herbivore gut microbiomes^20–22^. Anaerobic fungi possess unique root-like morphologies and enzyme complexes, termed fungal cellulosomes, that help localize lignocellulose-active enzymes to target polymers^23,24^. In addition, their genomes encode more carbohydrate-active enzymes (CAZymes) than industrial enzyme-producing fungi such as *Aspergillus* and *Trichoderma*^25,26^. A considerable fraction (~60–75%) of open reading frames in all sequenced Neocallimastigomycetes genomes are not functionally annotated because of the density of repeats, high adenine–thymine content and significant divergence from their closest known relatives^27,28^. Although challenging in many contexts, the unannotated gene content of Neocallimastigomycetes also provides an excellent opportunity to discover novel enzymes, particularly novel lignocellulose-active enzymes, for advancing sustainable biotechnologies^26,29,30^.

Undescribed interactions between anaerobic fungi and lignin constitute a major knowledge gap as Neocallimastigomycetes must at least circumvent lignin to access cellulose and hemicelluloses, their primary carbon sources. Previous efforts to interrogate interactions between ruminal microbes and lignin suggested that lignin might be directly or indirectly affected by anaerobic fungi, as implied by the accumulation of lignin degradation products^31–34^ or a reduction in lignification for processed plant material^35–40^. However, in the absence of further, direct evidence of lignin deconstruction, the observed release of monoaromatics from lignocellulose was attributed to hemicellulosic sidechain cleavage, a process anaerobic fungi facilitate^31,41^. Advances in two-dimensional heteronuclear single-quantum coherence nuclear magnetic resonance (2D-HSQC-NMR) spectroscopy allow direct examination of native lignin bonds in whole cell wall materials before and after the action of organisms or enzymes^42–45^. Recently, 2D-HSQC-NMR helped describe delignification by white rot^46^ and brown rot^47^ fungi, identified a novel ligninase produced by *Parascedosporium putredinis* NO1^48^ and suggested anti-lignin activity in consortia derived from termite microbiomes^49^. In this Article, we use a similar approach to determine that members of Neocallimastigomycetes deconstruct lignin during the degradation of plant biomass.

We demonstrate that Neocallimastigomycetes anaerobically deconstruct lignin. Compelling evidence is provided by using 2D-HSQC-NMR supported with gel-permeation chromatography (GPC) analysis for molecular mass, by using liquid chromatography-mass spectrometry (LC-MS) and by elucidating changes in the percentage composition of lignocellulose. Using RNA sequencing (RNA-seq), we further implicate a set of conserved anaerobic fungal genes, the products of which might be responsible for anaerobically deconstructing the constituent polymers of lignocellulose. The identified genes of interest are highly expressed in culture conditions containing complex lignocellulose, and we curate them using bioinformatic methods with predictive models to identify those most likely to encode novel lignocellulose-active enzymes.

## Comparing activity across carbon sources

**Fig. 1:**
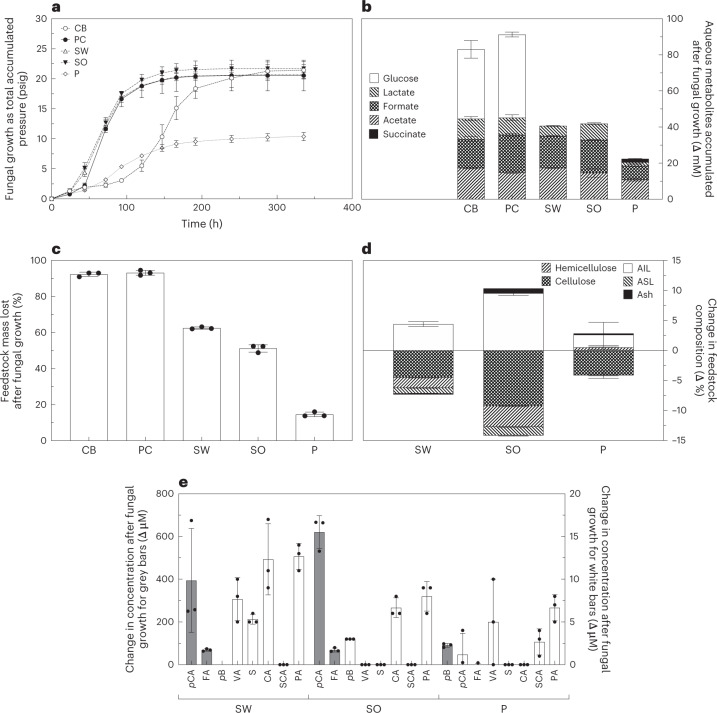
Anaerobic fungus *N. californiae* grows on, deconstructs and metabolizes a variety of lignocelluloses as well as other carbohydrate substrates. **a**, Fungal activity plotted as the pressure of fermentation gases accumulated by the fungus over time when grown on cellobiose (CB), purified cellulose (PC), switchgrass (SW), sorghum (SO) and poplar (P). **b**, The total change in metabolite concentrations, as measured by HPLC. **c**, The percentage of feedstock deconstructed after growth as measured by mass loss in the case of solid substrates and HPLC in the case of cellobiose. **d**, The change in percentage composition of each lignocellulose type. **e**, Monoaromatics present in fungal growth media at the end of fungal cultivation, as measured using LC-MS. FA, ferulic acid; S, syringic acid; CA, caffeic acid; SCA, salicylic acid. Panels **b**–**e** represent differences between inoculation and the final time point of **a** at 336 h. In all panels, error bars represent the standard deviation of biological replicates centred on the mean (*n* = 3).

The anaerobic fungus *Neocallimastix californiae* was cultivated on grass and wood lignocellulosic substrates (sorghum, switchgrass and poplar) with different lignin compositions. The metabolic activity of the fungus, the extent of lignocellulose solubilization, changes to lignocellulose composition and accumulation of lignocellulose degradation products in fungal growth media were measured (Fig. 1). Cultures of *N. californiae* produced fermentation products (Fig. 1a,b), depolymerized lignocellulosic biomass (Fig. 1c,d) and freed diverse aromatic monomers from lignocellulose (Fig. 1e). We measured no monoaromatics in the purified cellulose or cellobiose treatments indicating that the fungus did not produce detectable aromatic monomers from simple carbohydrates. The metabolic activity of the anaerobic fungus was comparable when grown on purified carbohydrates (cellulose and cellobiose) and grass (sorghum and switchgrass) lignocelluloses but was lower on poplar (Fig. 1a,b). Cultures grown on cellobiose exhibited an extended lag phase (Fig. 1a).

## Measuring lignocellulose deconstruction

**Extended Data Fig. 1:**
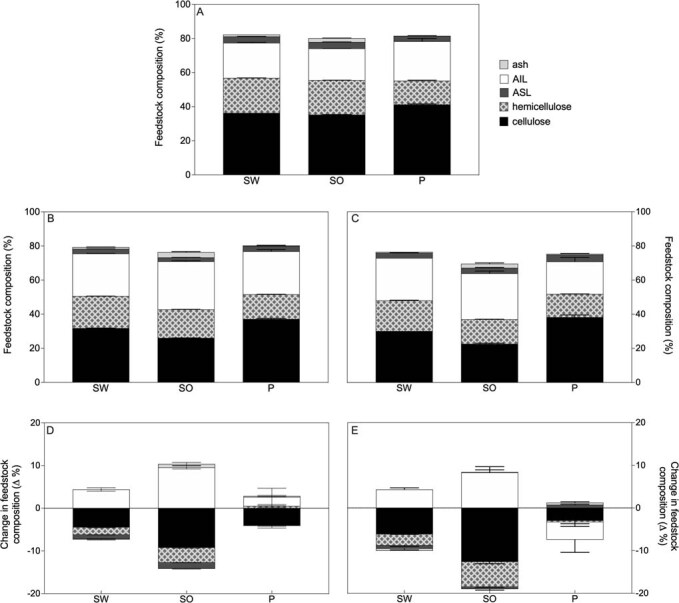
Biomass composition before and after growth shows selective removal of cellulose and hemicellulose, but also some acid-soluble lignin removal. In the figure legend, ASL abbreviates acid-soluble lignin, whereas AIL abbreviates acid-insoluble lignin. Panel A shows the starting composition of sorghum (SO), switchgrass (SW), and poplar P. Panel B represents the composition of SO, SW, and P after the growth of *Neocallimastix californiae*, and panel C represents the composition of SO, SW, and P after the growth of *Anaeromyces robustus*. Panel D shows the differences between panel B and panel A, and panel E shows the differences between panel C and panel A. Error bars in all panels represent the standard deviation of biological replicates (n = 3).

Compositional analyses determined the relative cellulose, hemicellulose, lignin and ash fractions of lignocellulose before and after fungal growth, providing a glimpse into the extent of deconstruction for the various polymers (Fig. 1d). The amount of cellulose and hemicellulose decreased for all lignocellulose types examined, and the proportion of acid-soluble lignin (ASL) also fell in grass lignocelluloses (Fig. 1d). Conversely, acid-insoluble lignin (AIL) made up a greater proportion of the total lignocellulose mass after growth. We further observed similar patterns of cellulose, hemicellulose and ASL loss for a second anaerobic fungal isolate, *Anaeromyces robustus*, confirming that observations of ASL reduction were not limited to a single isolate or genus (Extended Data Fig. 1). Remarkably, *A. robustus* reduced both ASL and AIL when grown on poplar, and this action contrasts with all other lignocellulose–fungus pairings (Extended Data Fig. 1).

**Fig. 2:**
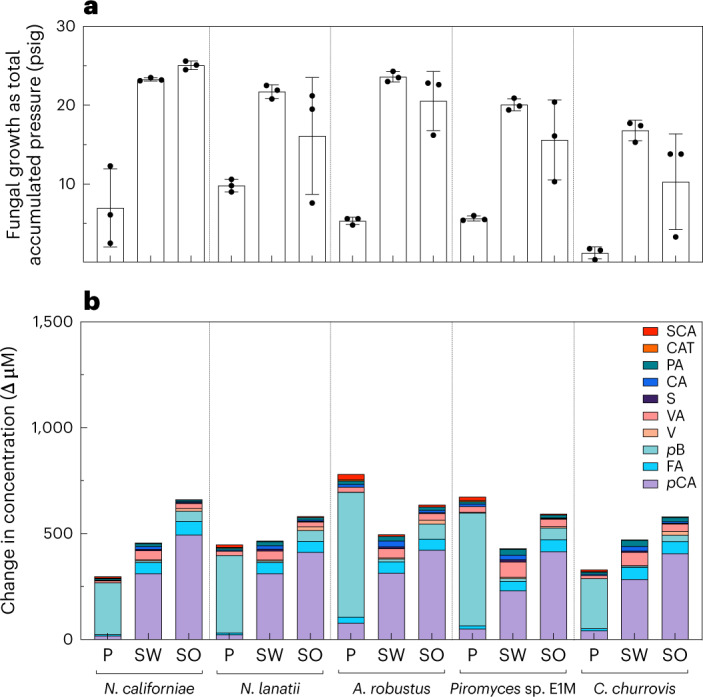
Anaerobic gut fungi from four genera solubilize diverse aromatic monomers from lignocellulose. Five strains of anaerobic gut fungi were grown on three different lignified substrates, and all released monoaromatic chemicals into the solution after growth. Lignocellulose types tested are poplar, switchgrass and sorghum. **a**, The total pressure accumulated over 10 days as a proxy for fungal growth. **b**, The mean difference between the concentration of various monoaromatics before and after fungal growth. Abbreviations for monoaromatic compounds are the same as in Fig. 1. CAT, catechol; V, vanillin. The vertical order of the legend matches the order of the stacked bars. As the growth medium is undefined, the values for uninoculated controls are subtracted from experimental values to calculate the values in **b**. The names of anaerobic fungi are as follows: *N. californiae*, *N. lanatii*, *A. robustus*, *Piromyces* sp. E1M and *C. churrovis*. The values shown in both panels are the means of biological replicates, and the error bars in **a** represent the standard deviations of these replicates (*n* = 3).

**Extended Data Fig. 2:**
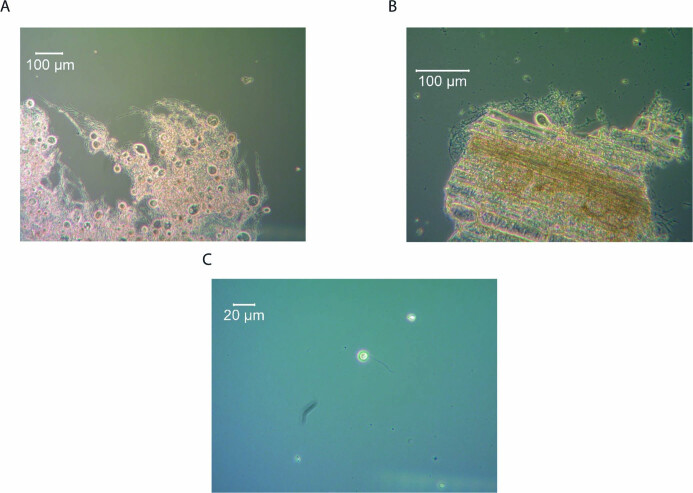
The novel anaerobic fungal isolate, *Piromyces* sp. E1M, possesses standard Neocallimastigomycetes morphology. These phenotypes are consistent with the genera *Piromyces* and *Neocallimastix*, and was classified as belonging to the genus *Piromyces* based on its internal transcribed spacer region (ITS1) and large ribosomal subunit (LSU) gene sequences. Mats of interlocking microrhizoids form when the fungus is cultivated on soluble sugar dimers, such as cellobiose, shown in panel A. Rhizoidal networks intercalate lignocellulose in panel B during fungal colonization. The fungus produces motile zoospores, as displayed in panel C. These are single observations and not necessarily representative of the average strain morphology.

The liberation of monoaromatics from lignocellulose was also observed in cultures of four additional isolates within Neocallimastigomycetes (Fig. 2). Cultures fed with grass lignocelluloses accumulated more than 450 µM of total measured monoaromatics, and the dominant chemical species was *p*-coumaric acid (*p*CA). Fungi liberated more variable concentrations of monoaromatics from poplar (200–750 µM), and the predominant chemical species freed was *p*-hydroxybenzoic acid (*p*B). All isolates accumulated greater than 200 µM *p*CA from grass lignocellulose or *p*B from poplar, and all other concentrations of monoaromatics liberated were less than 10 µM (Fig. 2b). Diverse fungi freed similar rank-ordered profiles of monoaromatics from sorghum and switchgrass, and the top five compounds released were consistent. In order of descending concentration, these compounds were *p*CA, ferulic acid, vanillic acid (VA), protocatechuic acid (PA, 3,4-dihydroxybenzoic acid) and syringic acid. Poplar–fungus pairings resulted in a different profile of monoaromatics, namely *p*B, *p*CA, VA, PA and salicylic acid, in order of decreasing concentration. Some isolates, specifically *A. robustus* and *Piromyces* sp. E1M (Supplementary Text and Extended Data Fig. 2), liberated ten times more *p*B from poplar than *Neocallimastix* isolates and *Caecomyces churrovis* (Extended Data Table 1).

**Fig. 3:**
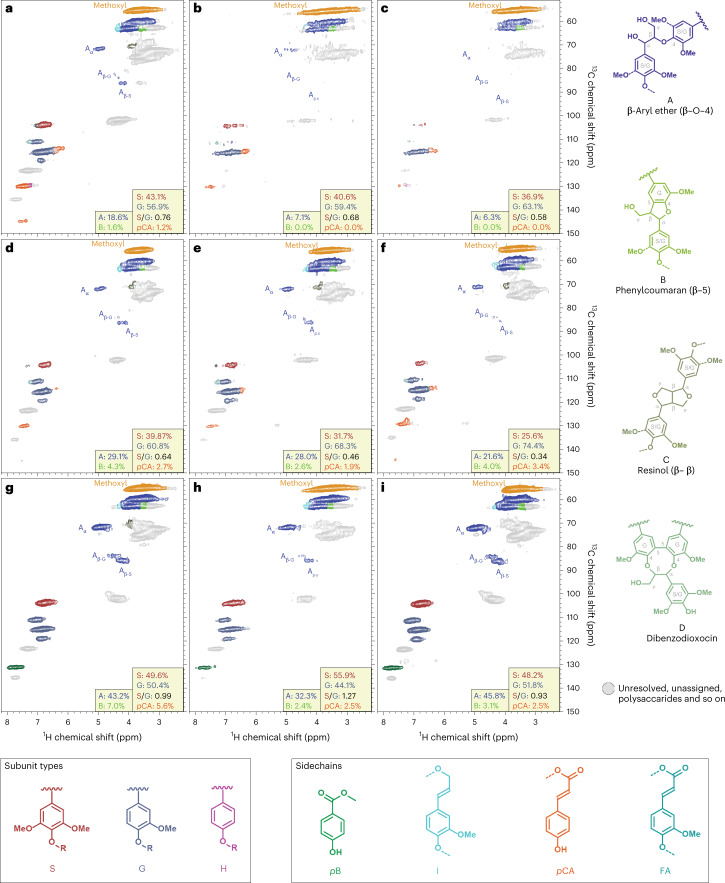
2D-HSQC-NMR data show that anaerobic fungi deconstruct aromatic and aliphatic regions in various lignins and remove lignin pendent groups. **a**, Sorghum uninoculated control. **b**, Sorghum after *N. californiae* growth. **c**, Sorghum after *A. robustus* growth. **d**, Switchgrass uninoculated control. **e**, Switchgrass after *N. californiae* growth. **f**, Switchgrass after *A. robustus* growth. **g**, Poplar uninoculated control. **h**, Poplar after *N. californiae* growth. **i**, Poplar after *A. robustus* growth. Inset values provide relative comparisons of the lignin components determined from contour volume integrals where S + G = 100% (right insets) or A + B = 100% (left insets). Lignin H units are below the detectable limit, as are resinol and dibenzodioxocin structures. The *p*CA and *p*B pendent ester fractions are calculated on a ‘core-lignin’ basis meaning the integral for the ester divided by S + G (pendent ester/S + G = %). Legends for monomeric lignin subunits, pendent esters and units with their characteristic inter-unit linkages are colour-coded to match their signals in the spectra.

**Extended Data Fig. 3:**
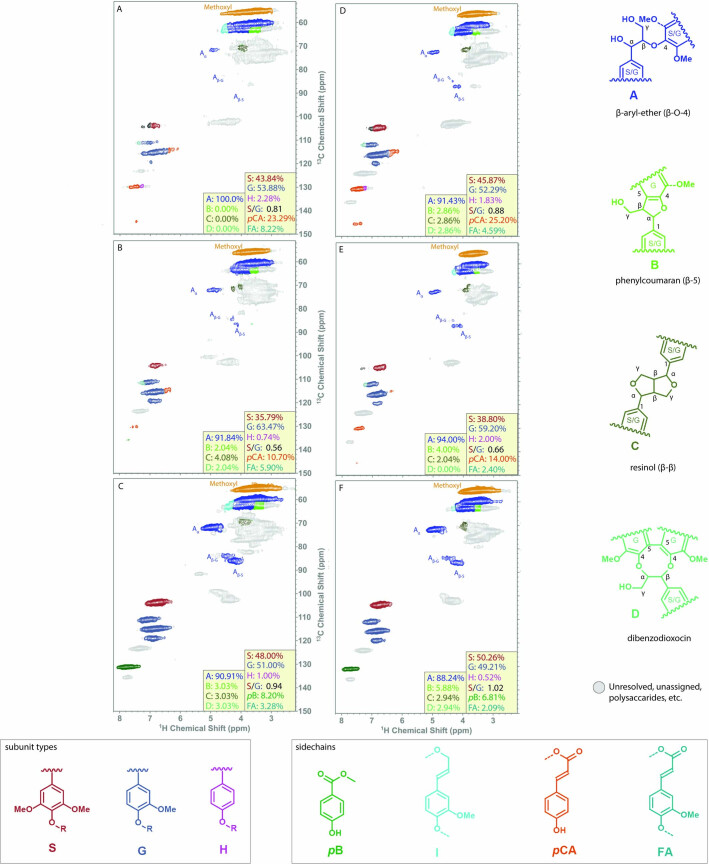
Two-dimensional-HSQC-NMR data for unautoclaved controls compared with uninoculated, autoclaved controls. The features in lignin shift during the process of sterilizing the growth medium, but these changes are markedly different from changes induced by fungi. Unautoclaved controls are shown in panel A for sorghum, panel B for switchgrass, and panel C for poplar. Uninoculated, autoclaved controls are shown in panel D for sorghum, panel E for switchgrass, and panel F for poplar. In sorghum and switchgrass, S:G ratios increased in the autoclave *versus* decreasing during fungal treatment. In poplar, the S:G ratio decreased after autoclaving versus increasing in cultures treated with anaerobic fungi. All integration percentages depicted here are calculated on a ΣSG basis.

We next used 2D-HSQC-NMR to determine any modifications to lignin by characterizing lignocellulose spectroscopically before and after the activity of Neocallimastigomycetes. To ensure that any observed lignin-modifying activity was not restricted to a single isolate, strain or genus, we chose to collect data from two different fungal isolates: *N. californiae* and *A. robustus*. We found that both anaerobic fungal isolates deconstruct parts of lignin in diverse types of lignocellulose (Fig. 3, Extended Data Fig. 3 and Extended Data Table 2). Expected signatures such as hemicellulose re-modelling and the removal of *p*CA, ferulic acid and *p*B pendent units were apparent in the spectra of all feedstocks treated with anaerobic fungi. The lignin of uninoculated controls for sorghum, switchgrass and poplar exhibited varying S and G subunit content that shifted after incubation with the fungal isolates. The S:G ratios of the sorghum samples fell from 0.76 in the control sample to 0.68 when treated with *N. californiae* and 0.58 when treated with *A. robustus* (Fig. 3a–c). Similarly, both fungi reduced switchgrass S:G ratios from 0.64 in the uninoculated control to 0.46 after incubation with *N. californiae* and 0.34 after incubation with *A. robustus* (Fig. 3d–f). The selective removal of S subunits from sorghum, but not necessarily switchgrass and poplar lignins, coincides with a loss of β-aryl-ether (β–O–4-linked) units and phenylcoumarans (β–5). In sorghum, the almost complete depletion of these β–O–4s and β–5s provides strong evidence of extensive modifications to the lignin polymer. Selective removal of β-aryl-ether and phenylcoumaran units by fungi grown on switchgrass and poplar lignins is more limited than in sorghum, although altered distributions of these linkages are apparent. Changes to poplar contrasted with changes to grass lignocelluloses by resulting in higher S:G ratios in samples treated with *N. californiae* (1.27) and marginally lower with *A. robustus* (0.93) than in the uninoculated control (0.99) (Fig. 3g–i). It proved instructive to normalize the integration data on a methoxyl integral basis in addition to the S and G subunit (ΣSG) basis, even though this cannot clarify all features (Fig. 3 and Extended Data Table 2). Although methoxyl levels change with the S:G shifts, some alterations to S and G subunits appear to retain the methoxyl groups such that the S:G reductions and the losses of some lignin linkages (β–O–4 and β–5) in the grasses become more apparent when using this normalization.

**Extended Data Fig. 4:**
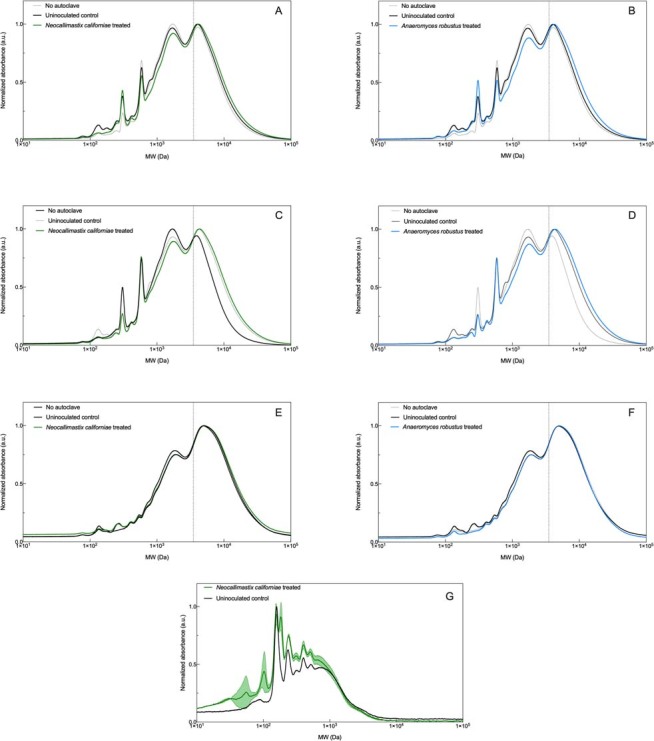
Gel-permeation chromatography (GPC) traces of lignin fragments, derived from lignocellulose or alkaline lignin. Lignocellulose derived samples are in panels A-F and alkaline lignin samples are in in Panel G. Traces show the molecular weight distribution of lignin oligomers before and after modification by cultures of anaerobic fungi and demonstrate changes to the molecular weight distribution of lignin oligomers. In all panels, the uninoculated and autoclaved control for the appropriate substrate is included (black, solid line). In panels A-F, the unautoclaved control is also included (black, dotted line). Vertical dotted lines in panels A-F indicate 3,500 Da on the x-axis. Panels A and B show lignin oligomers derived from sorghum after growth for *Neocallimastix californiae* A and *Anaeromyces robustus* B. Panels C and D show lignin oligomers derived from switchgrass after modification by *N. californiae* C and *A. robustus* D. Panels E and F show lignin oligomers derived from poplar after modification by *N. californiae* E and *A. robustus* F. Error bands shown in Panel G represent the standard deviation and the center line represents the mean calculated from n = 3 biological replicates.

Complementary GPC of lignin oligomers derived from the same lignocellulose fractions used to generate the NMR samples showed a loss of lignin oligomers under 3,500 Da after fungal modification but a relative increase of >3,500 Da lignin oligomers (Extended Data Fig. 4a–f). A subsequent GPC experiment, incubating *N. californiae* with alkaline lignin extract, also resulted in a reduction of molecular weight for lignin-derived oligomers, highlighted by the appearance of a new low-molecular-weight peak in the treated samples (Extended Data Fig. 4g).

## Identifying novel lignocellulose-active enzymes

**Fig. 4:**
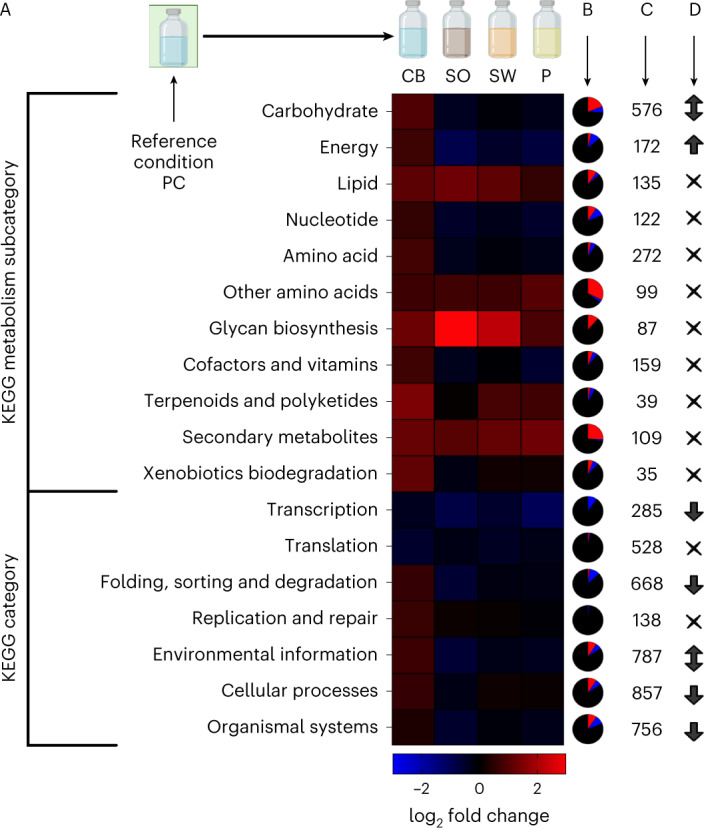
Differential regulation of KEGG-categorized *N. californiae* genes in response to lignocellulose availability reveals fungal transcriptome dynamics associated with carbohydrate metabolism. Lignocellulose or purified carbohydrate types: SO, SW and P. Genes were filtered for differential upregulation on SO, SW and P or differential downregulation on SO, SW and P as determined using DESeq2 with PC as the reference condition. Heat mapping in A represents the log_2_ fold change for transcripts in each KEGG category, calculated from all the gene length normalized (transcripts per million base pairs) values meeting statistical regulation criteria (*q* < 0.05). Pie charts in column B depict the fractions of genes for each KEGG category that were differently upregulated (red), downregulated (blue) or not differentially regulated on SO, SW and P (black). Counts in column C indicate how many genes are classified into each KEGG category in the *N. californiae* genome. Symbols in column D represent the results of a series of Fisher’s exact tests, where bidirectional arrows indicate a KEGG category was both significantly downregulated and upregulated, and unidirectional arrows indicate that the category was significantly upregulated or downregulated by SO, SW and P availability, whereas cross symbols represent no significant changes in category regulation. *P* values from Fisher’s exact tests were adjusted using the Benjamini–Hochberg correction, and errors are calculated from biological replicates (*n* = 3).

**Fig. 5:**
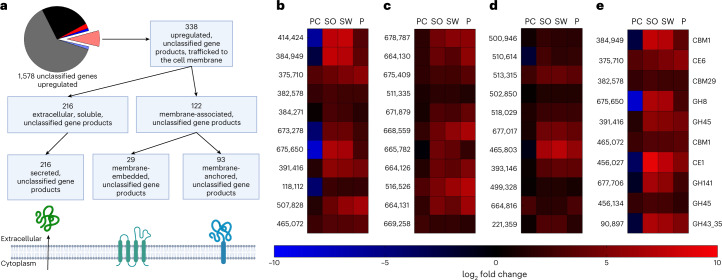
Predictive models implicate some unclassified *N. californiae* genes as novel lignocellulose-active enzymes. **a**, Unclassified genes presented here are upregulated (*q* < 0.05) on all three lignocellulose types relative to a purified cellulose control. Several subsets of the uncharacterized, upregulated genes are trafficked to the outer membrane of fungal cells as predicted by the workflow. In the pie chart, red, blue and black slices represent annotated genes that were upregulated, downregulated or not regulated; salmon, light blue, and grey slices represent unannotated genes that were upregulated, downregulated or not regulated. Signal peptides were predicted by SignalP5.0, and transmembrane helices were predicted by TOPCONS. **b**–**d**, Heatmaps for the ten most transcribed gene products of interest are shown for secreted soluble proteins (**b**), membrane-associated proteins having a single transmembrane helix (**c**) and membrane-embedded proteins having more than one transmembrane helix (**d**). **e**, A heatmap of the ten most expressed genes that were also predicted to have CAZyme domains by various models. In **e**, the CAZyme domain predicted by dbCAN and RoseTTAfold is noted to the right of the heatmap row. Identifiers to the left of each row of heatmaps in **b**–**e** are protein IDs from JGI’s Mycocosm (https://mycocosm.jgi.doe.gov/mycocosm/home). The *q* values are calculated using DESeq2 with PC as the reference condition.

**Extended Data Fig. 5:**
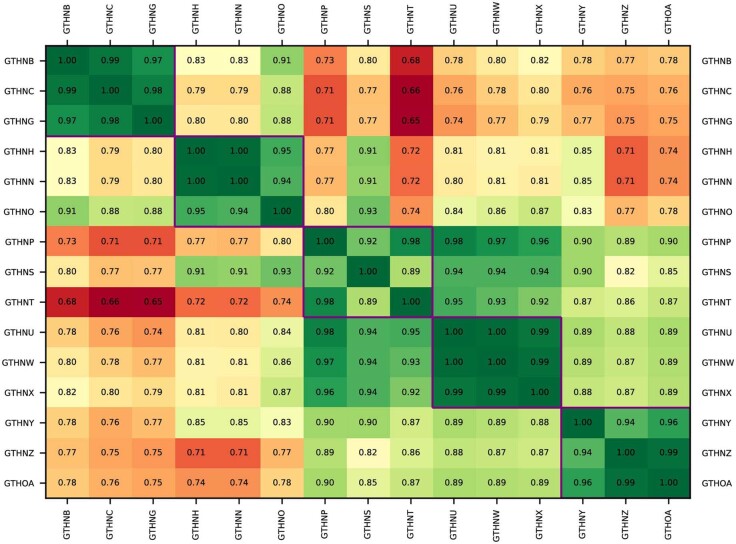
Pearson’s correlations of quality-controlled reads from each sequencing library suggest that biological triplicates are highly similar, and all should be included in subsequent differential expression analysis. As anticipated, lignocellulosic substrates induced more similar transcriptional profiles to one another than to purified cellulose or cellobiose, and grass (sorghum and switchgrass) lignocellulose treatments were more like one another than to poplar. Libraries are ordered as groups of replicates. Values in cells are Pearson’s correlation coefficients. Purple borders around replicate comparisons indicate acceptable correlation for continued analysis. Each identifier represents a single replicate. Samples correspond to the following identifiers: GTHNB = cellobiose replicate 1, GTHNC = cellobiose replicate 2, GTHNG = cellobiose replicate 3, GTHNH = purified cellulose replicate 1, GTHNN = purified cellulose replicate 2, GTHNO = purified cellulose replicate 3, GTHNP = switchgrass replicate 1, GTHNS = switchgrass replicate 2, GTHNT = switchgrass replicate 3, GTHNU = sorghum replicate 1, GTHNW = sorghum replicate 2, GTHNX = sorghum replicate 3, GTHNY = poplar replicate 1, GTHNZ = poplar replicate 2, and GTHOA = poplar replicate 3.

To identify what proteins might facilitate unknown aspects of anaerobic fungal lignocellulose deconstruction, we matched the deconstruction measurements to anaerobic fungal genes, upregulated in the presence of lignocellulose versus a purified cellulose control, using RNA-seq. The differential expression experiment examined *N. californiae* gene regulation on sorghum, switchgrass and poplar, comparing expression to control cultures growing on purified cellulose (Figs. 4 and 5, and Extended Data Fig. 5). Genes were considered of interest for this study if they were more expressed (*q* < 0.05)^50^ on sorghum, switchgrass and poplar. The most differentially regulated Kyoto Encyclopedia of Genes and Genomes (KEGG) categories^51^ meeting the specified conditions for functionally classified genes were metabolism, cellular processes and organismal systems (Fig. 4). Within the metabolism KEGG category, carbohydrate metabolism, energy metabolism and amino acid (AA) metabolism were the most impacted subcategories (Fig. 4).

The 25 most expressed and differentially upregulated CAZymes on each lignocellulose type were glycoside hydrolases (GHs) from various families. Six of the 25 most expressed, differentially upregulated CAZymes were shared across all three lignocellulose conditions, and all are annotated as diverse GHs; specifically, one representative was present from each of the GH9, GH1, GH3, GH27, GH32 and GH5 (subfamily one) classifications. None of the annotated carbohydrate esterases, enzymes associated with the cleavage of aromatic-hemicellulose ester linkages (CE1s), were differentially expressed in any condition. Of the 18 putative CE1s in the *N. californiae* genome, 11 had marginally increased transcript levels in all 3 lignocellulose-containing conditions, but none met the cut-off for statistically differential regulation.

Commonalities between the 25 most expressed and differentially regulated CAZymes in sorghum and switchgrass treatments distinguished these grass-induced CAZyme profiles from those of cultures grown on poplar. The transcriptomes from cultures grown on the two grass lignocelluloses shared most (19 out of 25) of their top 25 expressed CAZymes, but 13 of these 25 were not in the top 25 expressed CAZymes for the poplar treatment. All 13 genes prioritized in grass-induced transcriptomes but not poplar transcriptomes were various GHs (3 GH1s, 3 GH5s, 2 GH9s, 2 GH3s, 2 GH2s and 1 GH6). The occurrence of GH2s and GH6s and the presence of some carbohydrate-binding modules (CBMs), specifically fungal dockerin domains (CBM10s), were features of these grass-specific genes. The most upregulated and expressed genes in poplar transcriptomes were different, less diverse GHs. Ten of the top 25 expressed CAZymes specific to poplar were GH5s: 5 from subfamily 7, and 5 from subfamily 4. The other six uniquely poplar-associated genes were two glycoside transferases (GT8s), one polysaccharide lyase (PL1) and two GHs (GH26 and GH13) with predicted xylose binding modules (CBM35s).

We found 1,578 genes without functional classifications that had higher expression levels in the presence of lignocellulose versus the cellulose control and further curated this list with bioinformatic models (Fig. 5). Of the 1,578 identified genes without a KEGG classification, there were only 338 unannotated genes with both predicted signal peptides and increased expression levels across all three lignocellulose types (Fig. 5a). Of these, 29 had more than one transmembrane helix, 93 had exactly one transmembrane helix, whereas 216 had only a signal peptide and were hypothesized to represent extracellular and soluble proteins. Once filtered for a transcripts per million (TPM) cut-off of 10, only 179 out of 338 were identified as high-priority targets for characterization. Multiple sequences alignments completed on each subset of the 179 high-priority targets reveal some clusters of homologues within the gene sets (Extended Data Fig. 5)^52^. In addition, almost all predicted protein sequences for high-priority targets had partial homologues of >50% AA identity in one or more of the other sequenced anaerobic fungal genomes (Supplementary Table 1). In most cases, homologues were present across multiple genera of anaerobic fungi. Many of the predicted homologues from other genomes shared more than 90% AA identity, and most of these highly conserved peptide sequences were present across the entire diversity of available Neocallimastigomycetes genomes.

**Extended Data Fig. 6:**
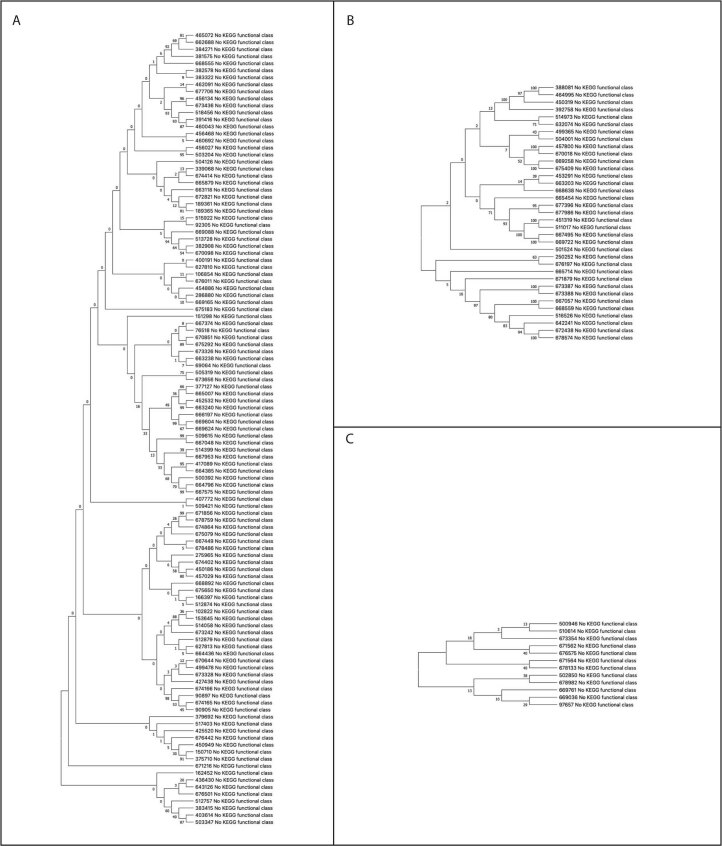
Multiple sequence alignments of gene products of interest reveal some clusters of predicted gene products with homologous regions. Only genes that had >10 TPM and *q* < 0.05 were included in these multiple sequence alignments. Each analysis was completed 100 times and the % of replicates in which the associated amino acid sequences clustered together are shown next to the branches. In panel A, a tree, built from predicted amino acid sequences for proteins with a signal peptide but no transmembrane, helices is shown. This tree contains 113 amino acid sequences and their alignment used 3613 amino acid positions. In panel B, a tree, built from predicted amino acid sequences for proteins with a signal peptide and a single terminal transmembrane, is shown. This tree contains 35 amino acid sequences and their alignment used 1845 amino acid positions. In panel C, a tree, built from predicted amino acid sequences for proteins with more than one transmembrane, is shown. This tree contains 12 amino acid sequences and their alignment used 1260 s amino acid positions. CAZyme domain predictions are included in cases only where predicted CAZymes clustered with other genes at a greater than 90% rate. Clusters of homologs have not been checked to see if regions of homology coincide with CAZyme active sites, however many genes cluster by predicted CAZyme active site.

Domain predictions helped curate high-priority genes of interest by implicating some unclassified, upregulated gene products in lignocellulose degradation. Of the 179 high-priority targets identified in this experiment, 29 of them contained CAZyme domains predicted by the database for automated carbohydrate-active enzyme annotation (dbCAN; Supplementary Table 2)^53^. Among the CAZyme domains most identified by dbCAN were those of putative acetyl xylan esterases, ferulic acid esterases, pectin acetyl esterases and diverse xylanases. Predictions from a three-track neural network that accounts for primary sequences and also considers atomic coordinates in folded proteins^54^ complemented and enhanced dbCAN predictions by finding the same CAZyme domains in all test cases in which dbCAN provided predictions (Supplementary Table 2 and Extended Data Fig. 5). The neural network analysis additionally identified several putative expansin-like proteins that may be involved in swelling plant cell walls, as well as a xylanase that was not predicted by dbCAN. Activity assays using lignin dimeric β-aryl-ether model compounds^48^ suggested that the anti-β-ether unit activity we observe in NMR spectra might be sensitive to environmental redox potential and non-enzymatic (Supplementary Text and Extended Data Fig. 6).

## Discussion

The 2D-HSQC-NMR data presented here provide evidence for the deconstruction of unpretreated naturally occurring lignin by cultures of anaerobic fungi. In all lignocellulose–fungus pairings, the S:G ratio of lignin after fungal growth was different than before, but the nature of that difference seemed to depend mainly on the lignin type. Sorghum and switchgrass lignins predictably had lower initial S:G ratios than the S-subunit-enriched poplar lignin. For grass lignins, fungal activity preferentially removed S subunits in sorghum, reducing S:G ratios by between 0.08 and 0.18, but in the case of poplar, S:G ratios increased by 0.28 in the *N. californiae* treatment but stayed about the same upon treatment with *A. robustus*. The magnitude of observed changes in S:G ratios is comparable to those reported for consortia enriched from termite microbiomes when incubated for 3 weeks with wheat straw^49^ and a brown rot fungus grown on aspen wood for 16 weeks^47^. Changes in S:G ratios are associated with the re-modelling of β-aryl-ether units (β–O–4 bonds) and phenylcoumarans (β–5 bonds) in the spectra we acquired. These shifts in S:G ratios are often also associated with disruption of resinols (β–β) and dibenzodioxocin (5–5/4–O–β bonds), but these units were not readily observed here. This activity against diverse bond types contrasts with recent findings in which activities were restricted to β-aryl-ether units, including those to tricin pendant units^48,49^. Non-specific activity against varied bond types suggests that indirect, small-molecule-mediated reactions could be responsible for some of the observed lignin bond cleavage. We hypothesize this action to be analogous to the way oxidative depolymerization processes in higher fungi operate non-enzymatically through the formation of aromatic acid radicals^6^.

The observations reported here can most accurately be summarized as lignin re-modelling accompanied by some lignin depolymerization, especially in the sorghum spectra. This extent of lignin deconstruction seems consistent with lignin removal from lignocellulose by anaerobic fungi to gain access to carbohydrate polymers. The most striking changes to lignin–lignin linkages we observed are in sorghum. In samples of sorghum, we observed complete elimination of phenylcoumaran signals and the drastic reductions of resonances corresponding to β-aryl-ether units after activity by both *N. californiae* and *A. robustus*. We also observed decreases of phenylcoumarans in all treated poplar and switchgrass samples, and the reduction of these signals in poplar samples was especially conspicuous. In both switchgrass samples and one poplar sample, β-aryl-ether units decreased in relative abundance after treatment. The observed changes extend to the core lignin polymer in the case of sorghum, but lignin deconstruction might be more dominated by phenolate (hydroxycinnamate and hydroxybenzoate) pendent groups in the cases of switchgrass and poplar.

The observed loss of β–O–4 and β–5 units in NMR spectra might be responsible for the lignin fragmentation we observed in GPC traces. The GPC data derived from the same lignocellulose samples as NMR spectra indicated a loss of lignin oligomers in the small to medium size range but a marginal accumulation of high-molecular-weight lignin oligomers. Incubations of *N. californiae* with alkaline lignin extract also resulted in fragmentation of lignin-derived oligomers as measured by GPC. These GPC observations, combined with the 2D-HSQC-NMR data, are consistent with the fragmentation of lignin into smaller oligomers but could indicate subsequent recondensation of lignin fragments. The NMR data, supported by fragmentation observed in GPC traces, show that Neocallimastigomycetes act on lignin itself through unknown means, which contrasts with the previously accepted hypothesis that changes to lignin caused by anaerobic fungi were incidentally induced by the cleavage of lignin–polysaccharide linkages.

The LC-MS and compositional analyses we conducted relate our novel lignin deconstruction observations to published results, and our measurements of monoaromatic accumulation and ASL loss agree well with previous observations of lignocellulose dissolution by anaerobic fungi. We hypothesize that the monoaromatics we observe in solution are the combined result of hemicellulose and lignin pendent ester hydrolysis and, in addition, lignin degradation, given the extent of deconstruction observed in NMR spectra. Many previous publications, using LC-MS or biomass compositional metrics to interrogate activity against lignin, fell short of being able to convincingly claim observation of anaerobic lignin modification by Neocallimastigomycetes^31,32,35–40^ because these methods cannot directly show reorganization of lignin–lignin linkages. The monoaromatics we observe in solution after fungal growth are of similar concentrations to those in previous observations and indicate that the isolates used in this study probably participate in lignocellulose deconstruction processes comparable to those of unidentified strains from previous reports^31,35^. Likewise, compositional analyses of the proportions of various polymers in lignocellulose, performed before and after fungal growth, indicated that the fungi in this study delignify plant cell walls in a manner consistent with literature reports^32,36,37,39,40^.

Anaerobic fungi have a predictable preference for depolymerizing cellulose, followed by a secondary preference for hemicelluloses, but they also reduce the percentage of ASL. The extent to which anaerobic fungi impact these polymers is consistent with their established preference for hexose sugars over pentose sugars and the removal of lignin to access carbohydrates^55^. In contrast to ASL, AIL increased in all pairings except one, a possible indication of lack of access to AIL moieties, the relative increase in AIL due to removal of cellulose and hemicellulose, or even recondensation, a well-documented phenomenon in lignin degradation processes^56^. Observed increases in the relative abundance of AIL in digested cell walls, combined with the selective elimination of β–O–4 signatures from sorghum NMR spectra, offer indications that the changes to the lignin we observe cannot result from hemicellulose modification and subsequent solubilization of peripheral, pendant lignin fragments alone. The almost complete removal of β–O–4 bonds implies that this bond type is specifically affected by the processes we observed as the lignin left behind in digested cell walls is more abundant but observably deficient in this specific feature. The primary conclusions we draw from the LC-MS and lignocellulose compositional change data are that our observations are consistent with literature reports, and the lignocellulose deconstruction we observe is probably conserved across the Neocallimastigomycetes class. These data speak to phenomena (dissolution and depolymerization) that could follow the lignin deconstruction we observe, but they do not establish anaerobic lignin deconstruction independently from the NMR observations.

The observed anaerobic lignin disruption by Neocallimastigomycetes contrasts with described aerobic processes in both completeness of lignin deconstruction and rate, particularly in sorghum. The lignin deconstruction processes we attribute to anaerobic fungi occur more quickly than those of other biological lignin deconstruction processes but do not reach the extent of deconstruction achieved by white rot fungi^6^. Characterization of anti-lignin processes by aerobic fungi typically occurs on the order of months^47^, whereas consortia derived from termite microbiomes^49^ took 33% longer to achieve less extensive lignin modification than that achieved by *N. californiae* and *A. robustus*. The accelerated nature of anaerobic fungal lignin deconstruction is logical in the context of herbivore digestion. This is the first report of a rumen microbe deconstructing lignin, but ruminant microbiomes extract nutritional value from lignocellulose on short timescales with limited residence times, and so anaerobic fungi might reorganize lignin rapidly to gain access to carbohydrates^32,38^.

Some of the upregulated, unannotated gene products we identified might be responsible for the observed anti-lignin chemistry attributed to anaerobic fungi, although many biological lignin deconstruction processes are non-enzymatic. An analysis of functionally classified *N. californiae* genes revealed expected regulation patterns, such as differential regulation of genes associated with carbohydrate metabolism in response to lignocellulose availability^20^. Furthermore, observations of the extended lag phase in the cellobiose condition generally agree with previous descriptions of catabolite repression in anaerobic fungi^57,58^. As there are no detectable homologues of known lignin-active enzymes in anaerobic fungal genomes, we hypothesize that observed lignin bond cleavage could be facilitated by the products of some of the currently unannotated genes that make up 72% of detectable open reading frames in the *N. californiae* genome^20,26^. The differential expression experiments and subsequent bioinformatic filtering identified 216 soluble, secreted proteins, as well as the 93 single transmembrane helix proteins that constitute gene targets for heterologous expression and characterization. We included proteins with one predicted transmembrane helix in this set of genes as some Neocallimastigomycetes lignocellulose-active enzymes, especially cellulosome complexes, are thought to be anchored to cell membranes via a single, terminal transmembrane helix^23^.

Several features of genes identified in the differential expression analysis reinforce the notion of characterizing them as encoding novel lignocellulose-active enzymes. Most of the genes of interest we identified are conserved across all sequenced Neocallimastigomycetes genomes. The ubiquity of these genes within a class of organisms that specializes in lignocellulose deconstruction indirectly supports the hypothesis that these unannotated genes might be involved in lignocellulose depolymerization. Furthermore, some AA sequences derived from these genes contain predicted lignocellulose-active enzyme domains identified using hidden Markov models^53^ or neural networks^54^. Among the enzyme domains most frequently identified are several classes of enzymes involved in hemicellulosic and hemicellulosic-substituent modification, including ferulic acid esterases, acetyl xylan esterases, pectin acetyl esterases and various xylanases. The bias of predictions towards domains that act on non-cellulose moieties is logical as control conditions in the differential expression experiment contained purified cellulose but no xylan or lignin and might allude to the importance of non-carbohydrate bond modifications in lignocellulose depolymerization by anaerobic fungi. The prevalence of uncharacterized CAZymes in this gene set indicates that our experimental design was effective at inducing the expression of uncharacterized lignocellulose-active enzymes and that novel lignin-active enzymes might eventually be identified within the genes of interest.

The biochemical mechanism by which Neocallimastigomycetes achieve anaerobic lignin deconstruction might be challenging to characterize, as was the case for aerobic lignin-active enzymes. Several aerobic lignin-active enzymes have only been described after decades of concerted efforts revealed their complex redox chemistry and non-enzymatic nature^6,9,59^. The re-modelling of multiple bond types within lignin polymers, as presented in our 2D-HSQC-NMR dataset, could be an indication that free radicals or other small molecules generated by anaerobic fungal enzyme systems non-specifically cause bond scission in lignin. Furthermore, the only measurable activity against lignin-mimicking model compounds we observed occurred in the small-molecule fraction of fungal supernatants, possibly implicating non-protein mediators in lignin deconstruction (Supplementary Text). Currently, only tentative hypotheses exist to explain how anaerobic microbes might be able to generate lignin-degrading radicals in the absence of oxygen^14–19^, and testing these hypotheses in Neocallimastigomycetes cultures and with heterologously expressed Neocallimastigomycetes proteins will be essential for unravelling the biochemical mechanism of anaerobic lignin deconstruction.

We expect this discovery that certain anaerobic organisms have the ability to deconstruct unpretreated, naturally occurring lignin will change perceptions of how lignocellulose can be processed, not only in ruminant guts but also in anaerobic environments throughout the biosphere^4,7,13^. Although the changes reported here are somewhat limited in comparison to those induced by previously described lignin depolymerizing organisms, such as white rot fungi, they are of broad significance as they alter understandings of lignin–anaerobe interactions that are commonplace in diverse environments. Removal of lignin allows anaerobic microbes access to labile carbon in carbohydrate polymers that can be directly remineralized to greenhouse gases or diverted into other processes such as animal metabolism. Furthermore, decarbonization biotechnologies, such as lignocellulosic biorefineries, could benefit from the increased availability of diverse, biological lignin deconstruction mechanisms that operate under anaerobic conditions^5,8^.

## Methods

### *N. californiae* cultivation conditions

To explore the possibility that anaerobic fungi deconstruct lignin, we completed primary experiments with the robust anaerobic fungus *N. californiae* that we previously isolated^20^. We measured the activity of *N. californiae* cultures on three types of lignocellulose and two purified carbohydrates. Switchgrass (*Panicum virgatum*) was obtained from the laboratory of D. Putnam at the University of California, Davis, whereas sorghum (*Sorghum bicolor*) and poplar (*Populus*) were obtained from the laboratory of G. L. Gresham of Idaho National Laboratory. All three lignocelluloses were ground using a Thomas-Wiley Mini Mill (Model 3383-L10, A. H. Thomas), and fragments were size-selected through a 2 mm screen by a vibratory sieve system (Endecotts). We chose the specified lignocellulose types because of their diverse lignin contents and their importance as feedstocks for biorefineries and livestock. Controls included purified cellulose (catalogue number 09-805, Fisher Scientific) and cellobiose (catalogue number A1455322, Fisher Scientific). Purified cellulose was cut into small strips (~3 mm) in lieu of milling.

*N. californiae* cultures for all primary experiments were grown in a minimalist version of Medium C described previously^22^. We supplied growth substrates (various lignocelluloses, cellulose or cellobiose) at 10 g l^−1^ in 80 ml serum bottles closed with butyl stoppers and aluminium crimp seals. Culture vessels had 100% CO_2_ headspaces, and cultures were 40 ml in liquid volume. Haem (protoporphyrin IX) and a vitamin solution were 0.22 µm filtered and added to cultures before inoculation as described and at the concentrations described previously^22,60^. Uninoculated controls for each feedstock were included alongside cultures of *N. californiae* to ensure that growth media were aseptic. Experimental cultures were grown in six replicates with three replicates earmarked for RNA collection and three replicates earmarked for endpoint analysis of *N. californiae* liquid metabolites, continued monitoring of fungal growth over time, and endpoint analysis of changes to lignocellulose. Samples for RNA-seq were collected 93 h after inoculation by combining the entire culture volume with RNA later in a 1:1 ratio (v/v).

At the final time point of this experiment (336 h after inoculation), liquid samples for high-performance liquid chromatography (HPLC) plus LC-MS were separated from solids (feedstock and fungal biomass) by centrifuging cultures at 16,000 × *g* for 20 min. Solids were resuspended in 10 ml of autoclaved MilliQ water to wash off salts and fungi and then re-pelleted at 16,000 × *g* for 20 min. Nine millilitres of water was removed, and solids plus the remaining 1 ml MilliQ were lyophilized for 24 h in a FreeZone 4.5 Liter Benchtop Freeze Dry System (part number 77500200, Labconco). These washed and dried lignocellulose samples were retained to measure changes to lignin and percentage lignocellulose composition.

### Measurement of *N. californiae* metabolic activity with HPLC and total pressure accumulation

As a proxy for fungal growth, total fermentation gas accumulation was monitored daily using the pressure transducer technique (PTT) as described previously^61,62^. After measuring daily pressure accumulation, headspaces were vented to 6.9 kPa.

We further quantified fungal metabolites as a secondary metric of fungal activity using HPLC as previously described^21,22^. Samples were run on a 1260 Infinity HPLC (Agilent Technologies) equipped with an Aminex HPX-87H analytical column (part number 1250140, Bio-Rad). Separation conditions were 0.6 ml min^−1^, at 50 °C and used a 5 mM sulfuric acid (H_2_SO_4_) mobile phase. The refractive index detector was set to 35 °C, and the variable wavelength detector was set to 210 nm. Guard columns were a 0.22 µm physical filter, followed by a Coregel USP L-17 guard cartridge (Concise Separations). Compounds monitored were acetate, formate, succinate, ethanol, lactate, cellobiose and glucose. All HPLC standards of 0.1%, 0.05% and 0.01% (w/v) were prepared in minimalist Medium C to account for media background as this medium contains sterilized and clarified rumen fluid^22^. During analysis, blank media chromatograms were subtracted from all standard and experimental chromatograms using OpenLab CDS analysis software (version 2.6, Agilent Technologies).

Samples and standards for HPLC were prepared by acidification to a concentration of 5 mM H_2_SO_4_, incubated for 5 min at room temperature and spun at maximum speed in a tabletop centrifuge for 5 min to pellet fungal cells, proteins and other debris. The acidified samples were removed from above the pellet and 0.22 µm filtered through a polyethersulfone (PES) membrane into HPLC vials with 300 µl polypropylene inserts.

### Lignocellulose compositional analysis before and after *N. californiae* growth

We assayed relative lignocellulose composition before and after growth to determine whether *N. californiae* preferentially removed certain polymers from lignocellulose using the National Renewable Energy Laboratory’s two-step acid hydrolysis analytical protocols^63^. All compositional analyses were conducted in technical duplicates. Briefly, 200 mg of biomass and 2 ml of 72% H_2_SO_4_ were incubated at 30 °C while shaking at 200 rpm for 1 h. The resultant slurry was diluted to 4% H_2_SO_4_ with 56 ml of deionized water for secondary hydrolysis at 121 °C for 1 h. The reaction was quenched by cooling the flasks to room temperature before removing the solids by filtration. The filtrate was used to determine glucan, xylan and ASL composition, while the AIL and ash amounts were ascertained from the solid residue.

Glucose and xylose concentrations were determined from the filtrate using an Agilent HPLC 1260 Infinity system equipped with a Bio-Rad Aminex HPX-87H column and a refractive index detector at 35 °C. A solution of sulfuric acid (4 mM) was used as the mobile phase with a flow rate of 0.6 ml min^−1^ and a column temperature of 60 °C. The amount of cellulose (glucan) and hemicellulose (xylan) was calculated from the glucose and xylose content multiplied by the anhydro correction factors of 162/180 and 132/150, respectively.

ASL was estimated by measuring the ultraviolet absorption of the acid hydrolysis supernatant at 240 nm using a Nanodrop 2000 ultraviolet–visible spectrophotometer (Thermo Fisher Scientific). AIL was quantified gravimetrically from the remaining solids after heating samples overnight at 105 °C to obtain the weight of AIL and ash and subsequently repeating this procedure at 575 °C for at least 6 h to obtain the weight of ash alone.

### LC-MS sample preparation and run conditions for quantification of monoaromatics

Samples for LC-MS were prepared by filtering *N. californiae* supernatants through 0.22 µm PES membranes, then through 3,000 Da molecular weight cut-off, PES, centrifugal filter units (12,000 × *g*, 30 min, 20 °C). The filtrate from the 3,000 Da centrifugal filter was diluted with one-part HPLC grade methanol (v/v). Analytical standards of aromatic compounds, samples and uninoculated controls were analysed using an Agilent Technologies HPLC-ESI-TOF-MS^64^. Calibration curves were used for the absolute quantification of each analyte of interest. The theoretical mass-to-charge ratios of the deprotonated analytes were used to identify the aromatics of interest. The aromatics of interest were *p*CA (163.040068), ferulic acid (193.050632), *p*B (137.024418), vanillin (151.040068), VA (167.034982), syringic acid (197.045547), caffeic acid (179.034982), PA (153.019332), catechol (109.029503) and salicylic acid (137.024418).

### Cultivation of *A. robustus* for generation of additional lignocellulose samples

To collect corroborating lignocellulose samples representing the activity of a second fungal isolate, we repeated a limited version of the primary experiment previously described for *N. californiae* with *A. robustus*^20^. The cultivation conditions matched those described for *N. californiae* in the ‘*N. californiae* cultivation conditions’ section. Cultures were vented to 6.9 kPa every other day to avoid inhibition of fungal growth. After 14 days, solid samples of processed lignocellulose were collected and washed as described in the ‘*N. californiae* cultivation conditions’ section. These washed and dried lignocellulose samples were retained to measure changes to lignin. They were also used to measure changes to lignocellulose caused by fungal activity as described in the ‘Lignocellulose compositional analysis before and after *N. californiae* growth’ section.

### Extending LC-MS observations to four additional Neocallimastigomycetes isolates

To extend observations of monoaromatics released from lignocellulose to other anaerobic fungal isolates, we grew five different strains of anaerobic fungi, representing four genera, on sorghum, switchgrass and poplar. We grew *N. californiae*^20^, *A. robustus*^20^, *Piromyces* sp. E1M (Supplementary Text and Extended Data Fig. 2), *Neocallimastix lanatii*^28^ and *C. churrovis*^65,66^ with each of the lignocellulosic feedstocks (sorghum, switchgrass and poplar), in triplicate and using Medium C^20,61^. These growth conditions were required instead of those described in the ‘*N. californiae* cultivation conditions’ section as not all isolates grow well in the minimal version of Medium C that was used for the primary experiment and subsequent generation of *A. robustus* lignocellulose samples. Cultures were otherwise as described previously. Lignocelluloses were loaded at 1% (w/v), cultivation media was amended with haem and vitamins as previously described^21,22,60^, and culture growth was monitored with PTT followed by venting to 6.9 kPa^61^. Samples from this experiment were prepared and run as described in the ‘LC-MS sample preparation and run conditions for quantification of monoaromatics’ section.

### Isolation and classification methods for the *Piromyces* sp. E1M strain

*Piromyces* sp. E1M was isolated from the faeces of an Asian elephant at the Santa Barbara Zoo as previously described^66,67^. Briefly, faeces were collected and diluted in Medium C under anaerobic conditions in the presence of chloramphenicol, and lignocellulose was supplied as a carbon source. After dilution and subsequent growth, observed as pressure production accompanied by a clumped matt of lignocellulose, fungi were diluted in an anaerobic roll tube and allowed to grow for 4 days. A single colony representing a clonal fungus was picked from the wall of the roll tube in an anaerobic chamber and reinoculated into Medium C with chloramphenicol^66^. The roll tube process was repeated three times to obtain pure cultures, alternating between rolling out single colonies for picking and growing liquid cultures in the presence of chloramphenicol.

The novel isolate was classified as belonging to the genus *Piromyces* on the basis of its internal transcribed spacer region (ITS1) and large ribosomal subunit (LSU) gene sequences. The ITS1 region of the genomes and the ribosomal RNA gene of the fungus were amplified with primers JB206/JB205, and a region of the large subunit 28 S rDNA was amplified using primers NL1/NL4^68^. Tentative taxonomy was assigned to the novel isolate using multiple sequence alignments conducted by the Ribosomal Database Project (RDP) Classifier^69,70^. The UNITE Fungal ITS database was used to classify the ITS1 sequence^71^, and the RDP Classifier Fungal 28 S database was used to classify the LSU sequence. The ITS and LSU sequences can be found in GenBank under BioProject accession number PRJNA800048.

### Whole plant cell wall 2D-HSQC-NMR sample preparation and data acquisition

Dried, washed lignocellulose samples for 2D-HSQC-NMR were collected from both *N. californiae* and *A. robustus* cultures. These lignocellulose samples were acted on by fungi, separated from liquid media and washed as described in the ‘*N. californiae* cultivation conditions’ and ‘Cultivation of *A. robustus* for generation of additional lignocellulose samples’ sections. Lignocellulose (~200 mg) was ground with an MM300 Mixer Mill (Qiagen) using 2-mm-diameter stainless steel balls and a mixing frequency of 20 s^−1^. Samples were ground for 45 min in the cases of sorghum and switchgrass samples or 80 min for poplar samples. These conditions are not the fine ball milling specified in the original methods, but grinding times were selected to generate material with particle sizes that are optimal for gelling, according to ref. ^42^. The ground samples were prepared for 2D-NMR experiments as described previously^42,43^. In a 5 mm NMR tube, ~120 mg of ground plant material was added along with 1 ml of pre-mixed DMSO-*d*_6_/pyridine-*d*_5_ solvent (4:1, v/v) to swell the lignocellulose and form a gel. The NMR tubes were sealed and sonicated for 4 h with a 30 min interval every hour until the gel became apparently homogeneous.

The 2D-HSQC-NMR spectra from lignocellulose processed by *N. californiae* or *A. robustus* were collected on a Bruker Avance I 800 MHz spectrometer equipped with a Bruker Triple Resonance Probe (TXI) at 310 K. A standard Bruker pulse sequence (hsqcetgpsisp2.2) was used with parameters that are typical for lignocellulose samples. Data were acquired using Bruker’s TopSpin software (version 4.1.0). The HSQC spectra were collected from 11 ppm to −1 ppm in the *F*_2_ (^1^H) dimension with 1,024 data points and 53 ms acquisition time with an interscan pulse delay of 1 s, and from 165 ppm to −10 ppm in the *F*_1_ (^13^C) dimension with 256 data points and 3.5 ms acquisition time. For each evolution period (t1) increment, 256 scans were recorded. The central DMSO solvent peak was used as a reference for the chemical shift calibration for all samples (*δ*_C_ 39.5 ppm, *δ*_H_ 2.5 ppm). All HSQC spectra were processed using typical cosine-squared apodization in both *F*_2_ and *F*_1_ dimensions, and the contours were integrated using MestreNOVA (version 14, Mestrelab Research). Peaks were assigned according to published data^42,43,72,73^.

Two sets of controls were included in this NMR experiment to account for the unlikely possibility that lignin was inadvertently deconstructed by autoclaving lignocellulose in fungal growth media. These unautoclaved controls were treated identically to the experimental samples by washing them with MilliQ water and then freeze-drying them. As the spectra of unautoclaved controls were similar to the no-inoculum controls, we include the no-inoculum controls in the main text comparisons to treated samples as this comparison is more direct. Extended Data Fig. 3 shows comparisons of the no-autoclave controls and no-inoculum controls.

### Cultivation of *N. californiae* with added alkaline lignin extract

To investigate whether anaerobic fungi affect the size of lignin-derived extractives, we grew the anaerobic fungus *N. californiae* in M2 media with both cellulose and alkaline lignin. All cultures received 0.22 µm, PES-filtered amendments of vitamins and haem, as previously described^21,22^. Alkaline lignin (catalogue number L0082, TCI America) was dissolved in MilliQ water and added to a final concentration of 2.5 g l^−1^. Cultures were 40 ml in liquid volume and were supplied 10 g l^−1^ cellulose (catalogue number 09-805, Fisher Scientific) as a primary carbon source. Experimental cultures were grown in triplicate, and an uninoculated control, with the same 0.22 µm filtered amendments, was included. At the cessation of growth, as measured by PTT^61^, bottles were collected, and solids were separated from supernatants by centrifugation (5,000 × *g*, 5 min). Supernatants were then freeze dried for 48 h in a FreeZone 4.5 Liter Benchtop Freeze Dry System (part number 77500200, Labconco). Alkaline lignin retrieved from these freeze-dried samples was then processed for GPC.

### GPC for lignin oligomer size

GPC was used to determine the relative molecular weight distribution of lignin in lignocellulose before and after treatment with *N. californiae* or *A. robustus*^74^. These lignocellulose samples were the same as those processed for NMR in the ‘Whole plant cell wall 2D-HSQC-NMR sample preparation and data acquisition’ section. Following standard protocols, 10 mg of lignocellulose was incubated in 2.5 ml of acetic acid and acetyl bromide (92:8) and stirred at 50 °C for 2 h to dissolve the lignin. Excess acetyl bromide and acetic acid were removed with a rotary evaporator connected to a high-vacuum pump and a cold trap. Acetylated lignin was immediately dissolved in tetrahydrofuran and filtered through 0.2 µm polytetrafluoroethylene filters. Samples of alkaline lignin from the experiments detailed in the ‘Cultivation of *N. californiae* with added alkaline lignin extract’ section were prepared for GPC analysis in the same way, but 10 mg of alkaline lignin was added to the acylation reaction instead of 10 mg of lignocellulose.

GPC analysis of both lignocellulose-derived lignin fragments and alkaline lignin fragments was conducted using an Ecosec HLC-8320GPC (Tosoh) equipped with Agilent Technologies PLgel 5 μm Mixed-D column and a Diode Array Detector. Tetrahydrofuran, spiked with 250 ppm of butylated hydroxytoluene, was used as the mobile phase with a flow rate of 1 ml min^−1^ and a column temperature of 40 °C. The GPC standards for oligomer size were polystyrene oligomers ranging from 162 g mol^−1^ to 29,150 g mol^−1^ (part number PL2013, Agilent Technologies).

### RNA extraction from *N. californiae* samples for differential expression analysis

To conduct differential expression analysis, samples of *N. californiae* for RNA, collected as described in ‘*N. californiae* cultivation conditions’ section, were lysed, and RNA was extracted. Samples were pelleted at 16,000 × *g* for 20 min to separate RNAlater and cultivation medium from solids, then solids were flash frozen in liquid nitrogen and stored at −80 °C until extraction. Fungal pellets were ground to a fine powder in liquid nitrogen using a mortar and pestle, and purification of RNA followed the Qiagen RNAeasy kit (part number 74004, Qiagen) protocol, including homogenization with a QIAshredder column (part number 79656, Qiagen) and an on-column DNAse digestion (part number 79254, Qiagen). The quality of extracted RNA was assessed using a TapeStation microfluidic electrophoresis device equipped with RNA tape (part number 5067–5576 and 5067–5577, Agilent Technologies), whereas RNA quantity was assessed using a Qubit fluorometer (Thermo Fisher Scientific) and the RNA broad range assay (part number Q10211, Thermo Fisher Scientific).

### RNA library construction, sequencing, read quality control and alignment of reads to the genome

Sequencing libraries were created and quantified at the Joint Genome Institute (JGI). Stranded complementary DNA libraries were generated using the Truseq Stranded mRNA Library Prep (catalogue number 20020595, Illumina). Messenger RNA was purified from 200 ng of total RNA using magnetic beads containing poly-T oligos (catalogue number 20020595, Illumina). Purified mRNA was then fragmented using divalent cations (catalogue number 20020595, Illumina) and incubation at 94 °C for 2 min. The fragmented RNA was reverse transcribed using random hexamers and Super Script II enzyme (catalogue number 18064-022, Thermo Fisher Scientific), followed by second-strand synthesis. The fragmented cDNA was treated with end-pair, A-tailing, adapter ligation and 10 cycles of PCR. The prepared libraries were then quantified using KAPA Illumina Library Quantification Kit (Roche) and run on a LightCycler 480 real-time PCR instrument (Roche). The quantified libraries were multiplexed, and the pool of libraries was prepared for sequencing on the Illumina NextSeq 500 sequencer using NextSeq 500 High-Output Sequencing kit v2 (Illumina) and following a 2 × 150 indexed run protocol. This sequencing run resulted in 15 sequence libraries totalling 396 million 2 × 150 reads before quality control (QC). Raw reads of differential expression data are deposited in NCBI’s Sequence Read Archive and can be found with NCBI Sequence Read Archive accession numbers SRP288871–SRP288885.

We filtered and trimmed raw reads using a QC pipeline developed by JGI. Raw reads were assessed for artefact sequences with kmer matching, and detected artefacts were trimmed from the 3′ ends of the reads (kmer = 25, 1 mismatch allowed) using BBDuk (version 38.90)^75^. Reads matching RNA spike-ins, PhiX reads and reads containing any ambiguous nucleotides were removed. We performed quality trimming using the phred trimming method set to Q6. Finally, reads under a length threshold of 25 bases or that ended QC as less than one-third of the original read length were removed. This QC pipeline resulted in 235 million reads that were of high enough quality to align to the *N. californiae* reference genome.

We used raw gene counts to evaluate the level of correlation between biological replicates. Pearson’s correlations were calculated between replicates to determine which replicates could be used in the differential expression analysis. For all five treatments, all three biological triplicates were included in differential expression analyses as these replicates showed high correlation coefficients ranging from 0.89 to 1.00 (Extended Data Fig. 5).

Filtered reads from each library were aligned to the reference genome using HISAT2 version 2.1.0^76,77^. Strand-specific coverage bigWig files were generated using deepTools version 3.1^78^. FeatureCounts (version 2.0.0)^79^ was used to generate the raw gene counts file using an appropriate genome feature file (Neosp1_GeneCatalog_genes_20170918.gff). On average, 92.6% of quality-controlled reads in each sequence library were mapped to the reference genome. We only included primary hits assigned to the reverse strand in the raw gene counts.

### Differential expression analysis of *N. californiae* transcriptomes

We determined which genes were differentially expressed between the purified cellulose control condition and other culture conditions using DESeq2 (version 1.28.1)^50^. We used an adjusted *P* value of *q* < 0.05 as the statistical cut-off for labelling a gene as differentially upregulated or downregulated. Genes were included as genes of particular interest if they were upregulated on all three lignocellulosic substrates compared to purified cellulose, and they were not assigned a KEGG functional class.

Characterized and annotated genes were labelled with KEGG categories on the basis of assigned KEGG Orthology (KO) numbers retrieved from JGI’s Mycocosm. The total relevant transcripts for each KEGG category were determined by summing the total transcripts aligned to all differentially expressed genes within a given KEGG category for both the control condition and each treatment condition. We then calculated a log_2_ fold change for each KEGG category by log_2_ transforming the ratio obtained from dividing experimental transcripts by purified cellulose control transcripts. Gene counts for genes with multiple KO numbers, or KO numbers that reference multiple KEGG categories, were included in calculations for all categories they were classified in, but gene transcript counts were only included once per KEGG category even if they were mapped to a KEGG category more than once by multiple KO numbers. All parsing of differential expression data was conducted in Python 3.9.13, using tools from Biopython (version 1.79) and NumPy (version 1.23.1)^80–82^.

To describe the most drastically upregulated CAZymes, identified in each lignocellulose versus cellulose comparison, we selected the top 25 genes, determined by their mean TPM value for the treatment condition. These selected genes were differentially expressed in each condition and had been labelled with the KEGG category ‘Carbohydrate Metabolism’. We then retrieved protein sequences for these 576 genes and subjected them to the dbCAN2 classifier^53^ to assign CAZyme families and subfamilies to the CAZyme. After applying dbCAN2 annotations, the top 25 genes having the largest TPM allocations were compared to one another to determine similarities and differences in the most expressed CAZymes between conditions. Annotated CAZymes data were parsed in Python 3.0, using tools from Biopython and NumPy^80–82^.

### Using predictive bioinformatic tools to characterize genes of interest

Open reading frames in the *N. californiae* genome that are not functionally characterized and that were upregulated on all three lignocellulose types were further analysed using several predictive models. We used SignalP5.0 to determine whether genes of interest were likely to be trafficked to the exterior of the cell^83^. Likewise, we used the TOPCONS web server to predict whether gene products were likely membrane associated or instead were soluble proteins^84^. We also predicted CAZyme domains in genes of interest using the dbCAN web server^53^. All output data files were matched to differential expression data and metadata with Python 3.0, using both the Biopython toolkit and NumPy^80–82^. Further analyses were completed on genes that were considered high priority for characterization because they had TPM values greater than 10 TPM and *q* values less than 0.05, and we refer to these now as sequences of interest.

Sequences of interest were further submitted to the Rosetta server, which used RoseTTaFold to generate protein structure predictions^54,85^. This analysis helped to ensure confidence in dbCAN^53^ predictions, and so sequences with predicted CAZyme domains were analysed as positive controls. When RoseTTaFold predictions had confidence greater than 0.4 as computed by DeepAccNet, the model with the lowest average error estimate was searched for local structural similarity against the PDB100 database and AlphaFold Protein Structure Database (https://alphafold.ebi.ac.uk/) using FoldSeek (https://github.com/steineggerlab/foldseek) in 3 Di/AA mode. The lowest *E* value matches for bacterial and fungal proteins were compiled to suggest putative functions for each sequence of interest.

Multiple sequences alignments were completed to help identify sequences of interest which might have homologous domains and therefore could participate in similar functions. The evolutionary histories for three sets of genes of interest (secreted, soluble and membrane associated) were inferred using the neighbour-joining method^86^, and this analysis was replicated 100 times^87^. We calculated evolutionary distances using the Jones, Taylor, Thornton matrix-based method in units of the number of AA substitutions per site^88^ and removed all ambiguous positions for each sequence pair (pairwise deletion option). Evolutionary analyses were conducted in MEGA X^52,89^.

### Preparation of protein and small-molecule fractions for β-ether model assays

Fungal cultures of *N. californiae* and *A. robustus* were grown on sorghum in a minimalist version of Medium C^22^. Fungal supernatants were separated from fungal cells by anaerobically centrifuging whole cultures at 16,000 × *g* for 3 min. Supernatants were then decanted anaerobically and 0.22 µm filtered with a PES filter. Fungal cell pellets were either anaerobically or aerobically shaken on a vortexer for 20 min in sterilized tubes with sterilized 0.5 mm zirconia-silicate beads (part number 11079105Z, Biospec) and 500 µl of sterile phosphate buffer (100 mM, pH 7.0). Both aerobic and anaerobic lysates were then 0.22 µm filtered (PES) to remove any intact cells or large cellular debris. Using 10 kDa molecular weight cut-off centrifugal filtration devices (part number VS0291, Sartorius), fractions for both supernatants and lysates were buffer exchanged twice with anaerobic or aerobic phosphate buffer (100 mM, pH 7.0). Centrifugal filtrations were conducted both aerobically and anaerobically. The filtrates from the initial filtration, as well as the concentrated buffer, exchanged protein fraction from both cell lysate and supernatant, were retained.

To contrast the activity of fungal protein preparations in anaerobic and aerobic incubations, as described above, phosphate buffers (100 mM, pH 7.0) with and without oxygen were prepared. Aerobic phosphate buffer was prepared from MilliQ water and then stirred at room temperature for 1 week with a gas-permeable cover. Anaerobic phosphate buffer was prepared by boiling MilliQ water before passing that boiling water into an anaerobic chamber (atmosphere, 75% N_2_, 20% CO_2_, 5% H_2_) and dissolving phosphate salts after cooling. The anaerobic phosphate buffer was also stirred continuously for 1 week with a gas-permeable cover before being used in subsequent incubations. Before incubations, all phosphate buffers were 0.22 µm filtered. For small-molecule (<10 kDa) incubations, 200 µl of the desired phosphate buffer (aerobic or anaerobic) was added to each incubation.

### Activity assays for β-ether model compounds

We incubated β-ether model compounds with the fractions prepared in ‘Preparation of protein and small-molecule fractions for β–O–4 model assays’ section to reproduce the anti-lignin bond activity in vitro^48^. Each reaction was sampled five times before and after incubation, and fluorescence was read on a Tecan M200 plate reader (Tecan). For all assays, model compounds were supplied at 20 mM, and reaction buffers were phosphate buffers (100 mM, pH 7.0). Time-zero measurements were conducted immediately after adding model compounds to incubations, and time final measurements were taken after incubation of the model compounds in the reaction mixture for 24 h at 39 °C.

We added 50 µl of glycine buffer (pH 10.1, 100 mM) to 100 µl of reaction mixture to obtain fluorescence measurements. The mixture’s fluorescence was read in a black 96-well plate using an excitation wavelength of 372 nm and an emission wavelength of 445 nm, which correspond to the excitation and emission maxima for 4-methylumbelliferone.

1 $$\begin{array}{l}{\mathrm{Normalized}}\;{\mathrm{activity}}\\ = \displaystyle\frac{{\begin{array}{l}\left( T_{\mathrm{final}}\;{\mathrm{sample}}\;{\mathrm{fluorescence}}\;-\;T_{\mathrm{0}}\;{\mathrm{sample}}\;{\mathrm{fluorescence}} \right)\\ - \left({T_ {\mathrm{final}}}\; {\mathrm{blank}}\;{\mathrm{fluorescence}}\;-\; T_{0}\; {\mathrm{blank}}\;{\mathrm{fluorescence}} \right)\end{array}}}{{{{\upmu}}\,{\mathrm{g}}\;{\mathrm{of}}\;{\mathrm{protein}}\;{\mathrm{in}}\;{\mathrm{reaction}}}}\end{array}$$

The anti-probe activity in each incubation, in arbitrary fluorescence units, was calculated by subtracting the time-zero fluorescence measurements (*T*_0_ sample and blank fluorescence) from the time final measurements (*T*_final_ sample and blank fluorescence) and then dividing the change in fluorescence by the protein content of the incubation (μg of protein in reaction) (equation (1)). The changes in fluorescence for blank incubations, consisting of model compounds in phosphate buffer with no protein, were subtracted from all experimental assay values (equation (1)). Positive controls were tyrosinase from button mushroom (part number T3824, Sigma Aldrich), supplied at a concentration of ~35 µg purified protein per 1 ml reaction. The amount of protein in experimental treatments was determined using the Pierce Coomassie Plus (Bradford) Assay Reagent (part number 23238, Thermo Fisher Scientific). The uncertainties associated with time-zero means and the blank reaction means are propagated to the errors associated with the final normalized activity values, while protein concentrations are treated as constants.

### Reporting summary

Further information on research design is available in the Nature Portfolio Reporting Summary linked to this article.

## Supplementary information

Supplementary Information Supplementary text describing isolation of the *Piromyces* sp. E1M strain and activity assays using model compounds.

Reporting Summary

Supplementary Tables Supplementary Table 1: A table of high-priority genes of interest from this study, determined by combining differential expression with lignin deconstruction observations and predictive modelling. Genes of interest for this study are those that meet our criteria of upregulation on all three lignocelluloses and possession of a signal peptide. Supplementary Table 2: A table of the identified, upregulated genes of highest interest for this study that are conserved across the Neocallimastigomycetes taxonomic class. These results demonstrate that homologues of many gene products of interest are present in every available, sequenced anaerobic fungal genome, and some of these predicted peptides are highly conserved.

## Figures and Tables

**Extended Data Fig. 7:**
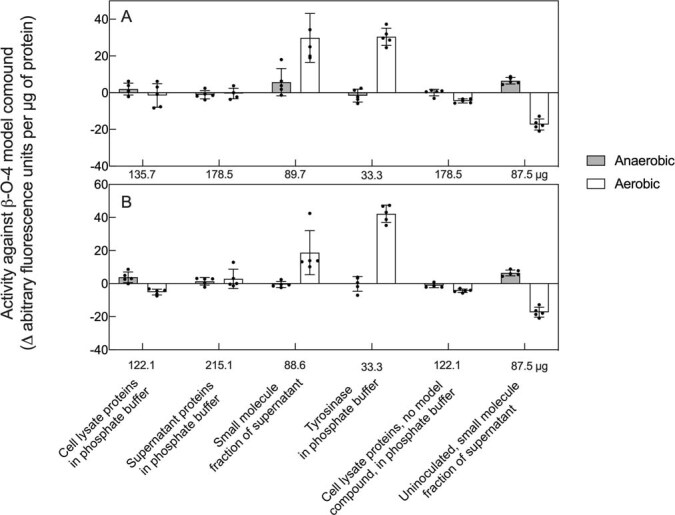
Model compound assays suggest lignin bonds are broken by small molecule-mediated redox reactions in Neocallimastigomycetes cultures. Data from *Neocallimastix californiae* cultures are depicted in panel A, whereas data from *Anaeromyces robustus* cultures are depicted in panel B. Values derived from blank incubations (with only model compounds and phosphate buffer) were subtracted from all depicted values. Tyrosinase incubations are positive controls. Cell lysate incubations without model compound added are included to account for any autofluorescence of fungal lysate. Error bars represent standard deviations for n = 5 unpaired technical replicates, where the uncertainty associated with blank TimeZero, blank TimeFinal, sample TimeZero, and sample TimeFinal have been propagated to calculate representative standard deviations. The change in fluorescence over 24 h is further normalized to the amount of protein in each incubation, and the protein concentration is shown below the x-axis for each incubation.

**Extended Data Table 1:**
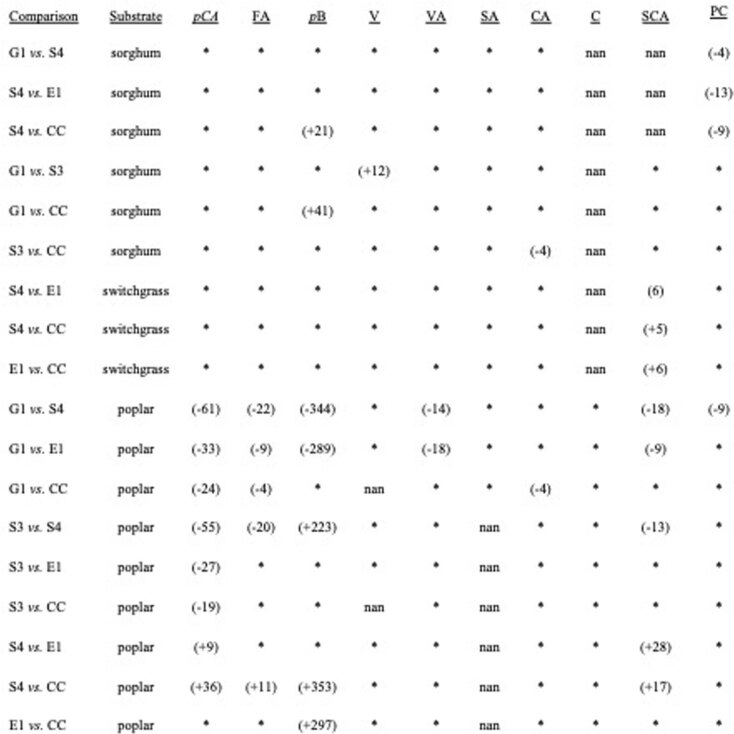
Some strains of anaerobic gut fungi more readily solubilize monoaromatics from lignocellulose than others, and this effect varies by substrate-fungus pairing

This table represents a series of *t*-tests conducted using the two-tailed Holm–Šídak method of comparison. In each cell, an asterisk represents *P* > 0.01, but if *P* < 0.01, the magnitude of the difference (in average µM concentration change) is given in parentheses. Means and associated errors for *t*-tests are calculated from biological replicates (*n* = 3). For magnitudes, values are positive if A > B in the comparison A versus B. Comparisons that are not shown had no significant differences. Shorthand abbreviations of strains are: G1 = *Neocallimastix californiae*, S3 = *Neocallimastix lanatii*, S4 = *Anaeromyces robustus*, CC = *Caecomyces churrovis*, E1 = *Piromyces* sp. E1M.

**Extended Data Table 2:**
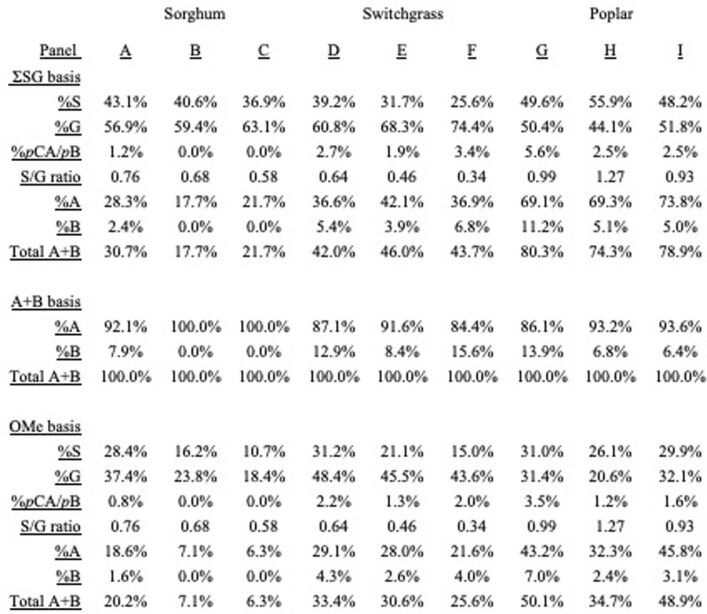
Relative amounts of subunits, sidechains or units derived from 2D-HSQC-NMR contour volume integrals

Contour volume integrals are normalized in three distinct ways: first using an S and G subunit basis where S = G = 100% (ΣSG basis), then on an A + B basis where A + B = 100% (A + B basis), and lastly on methoxyl basis where the volume of the methoxyl integral is considered as 100% (Ome basis).

## Data Availability

All data are available in the main text, the Extended Data, the Supplementary Information or through JGl’s Mycocosm (https://mycocosm.jgi.doe.gov/mycocosm/ home). Raw reads of differential expression data are deposited in NCBl’s Sequence Read Archive database and can be found with SRA accession numbers SRP288871–SRP288885. Amplicon sequences for fungal taxonomic classification are deposited in GenBank under BioProject accession number PRJNA800048. The CAZy database (http://www.cazy.org/), used for active site predictions, and the AlphaFold protein database (https://alphafold.ebi.ac.uk/), used for protein structural predications, are both publicly available. Strains and materials are available from the corresponding author upon request.

## References

[1] Bomble, Y. J. et al. Lignocellulose deconstruction in the biosphere. *Curr. Opin. Chem. Biol.* **41**, 61–70 (2017). 29100023 10.1016/j.cbpa.2017.10.013

[2] Ralph, J. et al. Lignins: natural polymers from oxidative coupling of 4-hydroxyphenyl-propanoids. *Phytochem. Rev.* **3**, 29–60 (2004).

[3] Liu, Q., Luo, L. & Zheng, L. Lignins: biosynthesis and biological functions in plants. *Int. J. Mol. Sci.* **19**, 335 (2018). 29364145 10.3390/ijms19020335 PMC5855557

[4] Robinson, J. M. Lignin, land plants, and fungi: biological evolution affecting Phanerozoic oxygen balance. *Geology* **18**, 607–610 (1990).

[5] Ragauskas, A. J. et al. Lignin valorization: improving lignin processing in the biorefinery. *Science* **344**, 1246843 (2014). 24833396 10.1126/science.1246843

[6] Janusz, G. et al. Lignin degradation: microorganisms, enzymes involved, genomes analysis and evolution. *FEMS Microbiol. Rev.* **41**, 941–962 (2017). 29088355 10.1093/femsre/fux049 PMC5812493

[7] Floudas, D. et al. The Paleozoic origin of enzymatic lignin decomposition reconstructed from 31 fungal genomes. *Science* **336**, 1715–1719 (2012). 22745431 10.1126/science.1221748

[8] Beckham, G. T., Johnson, C. W., Karp, E. M., Salvachúa, D. & Vardon, D. R. Opportunities and challenges in biological lignin valorization. *Curr. Opin. Biotechnol.* **42**, 40–53 (2016). 26974563 10.1016/j.copbio.2016.02.030

[9] Pollegioni, L., Tonin, F. & Rosini, E. Lignin-degrading enzymes. *FEBS J.* **282**, 1190–1213 (2015). 25649492 10.1111/febs.13224

[10] Silva, J. P., Ticona, A. R. P., Hamann, P. R. V., Quirino, B. F. & Noronha, E. F. Deconstruction of lignin: from enzymes to microorganisms. *Molecules* **26**, 2299 (2021). 33921125 10.3390/molecules26082299 PMC8071518

[11] Schmitz, O. J. et al. Animals and the zoogeochemistry of the carbon cycle. *Science* **362**, eaar3213 (2018). 30523083 10.1126/science.aar3213

[12] Godfray, H. C. J. et al. Meat consumption, health, and the environment. *Science* **361**, eaam5324 (2018). 30026199 10.1126/science.aam5324

[13] Young, L. Y. & Frazer, A. C. The fate of lignin and lignin‐derived compounds in anaerobic environments. *Geomicrobiol. J.* **5**, 261–293 (1987).

[14] DeAngelis, K. M. et al. Evidence supporting dissimilatory and assimilatory lignin degradation in *Enterobacter lignolyticus* SCF1. *Front. Microbiol.* **4**, 280 (2013). 24065962 10.3389/fmicb.2013.00280 PMC3777014

[15] Chaput, G. et al. Lignin induced iron reduction by novel sp., *Tolumonas lignolytic* BRL6-1. *PLoS ONE* **15**, e0233823 (2020). 32941430 10.1371/journal.pone.0233823 PMC7497984

[16] Billings, A. F. et al. Genome sequence and description of the anaerobic lignin-degrading bacterium *Tolumonas lignolytica* sp. nov. *Stand. Genom. Sci.* **10**, 106 (2015). 10.1186/s40793-015-0100-3 PMC4653933 26594307

[17] Duan, J., Liang, J., Wang, Y., Du, W. & Wang, D. Kraft lignin biodegradation by *Dysgonomonas* sp. WJDL-Y1, a new anaerobic bacterial strain isolated from sludge of a pulp and paper mill. *J. Microbiol. Biotechnol.* **26**, 1765–1773 (2016). 27381334 10.4014/jmb.1602.02014

[18] Woo, H. L. et al. Draft genome sequence of the lignin-degrading *Burkholderia* sp. strain LIG30, isolated from wet tropical forest soil. *Genome Announc.* **2**, e00637-14 (2014). 24948777 10.1128/genomeA.00637-14 PMC4064042

[19] Woo, H. L. et al. Complete genome sequence of the lignin-degrading bacterium *Klebsiella* sp. strain BRL6-2. *Stand. Genom. Sci.* **9**, 19 (2014). 10.1186/1944-3277-9-19 PMC4273726 25566348

[20] Solomon, K. V. et al. Early-branching gut fungi possess a large, comprehensive array of biomass-degrading enzymes. *Science* **351**, 1192–1195 (2016). 26912365 10.1126/science.aad1431 PMC5098331

[21] Gilmore, S. P. et al. Top-down enrichment guides in formation of synthetic microbial consortia for biomass degradation. *ACS Synth. Biol.* **8**, 2174–2185 (2019). 31461261 10.1021/acssynbio.9b00271

[22] Peng, X. et al. Genomic and functional analyses of fungal and bacterial consortia that enable lignocellulose breakdown in goat gut microbiomes. *Nat. Microbiol.* **6**, 499–511 (2021). 33526884 10.1038/s41564-020-00861-0 PMC8007473

[23] Haitjema, C. H. et al. A parts list for fungal cellulosomes revealed by comparative genomics. *Nat. Microbiol.* **2**, 17087 (2017). 28555641 10.1038/nmicrobiol.2017.87

[24] Lillington, S. P. et al. Cellulosome localization patterns vary across life stages of anaerobic fungi. *mBio* **12**, e0083221 (2021). 10.1128/mBio.00832-21 PMC8262932 34061594

[25] Grigoriev, I. V. et al. MycoCosm portal: gearing up for 1000 fungal genomes. *Nucleic Acids Res.* **42**, 699–704 (2014). 10.1093/nar/gkt1183 PMC3965089 24297253

[26] Seppälä, S., Wilken, S. E., Knop, D., Solomon, K. V. & O’Malley, M. A. The importance of sourcing enzymes from non-conventional fungi for metabolic engineering and biomass breakdown. *Metab. Eng.* **44**, 45–59 (2017). 28943461 10.1016/j.ymben.2017.09.008

[27] Wilken, S. E. et al. Genomic and proteomic biases inform metabolic engineering strategies for anaerobic fungi. *Metab. Eng. Commun.* **10**, e00107 (2020). 31799118 10.1016/j.mec.2019.e00107 PMC6883316

[28] Wilken, S. E. et al. Experimentally validated reconstruction and analysis of a genome-scale metabolic model of an anaerobic Neocallimastigomycota fungus. *mSystems* **6**, e00002-21 (2021). 33594000 10.1128/mSystems.00002-21 PMC8561657

[29] Podolsky, I. A. et al. Harnessing nature’s anaerobes for biotechnology and bioprocessing. *Annu. Rev. Chem. Biomol. Eng.* **10**, 105–128 (2019). 30883214 10.1146/annurev-chembioeng-060718-030340

[30] Lankiewicz, T. S., Lillington, S. P. & O’Malley, M. A. Enzyme discovery in anaerobic fungi (Neocallimastigomycetes) enables lignocellulosic biorefinery innovation. *Microbiol. Mol. Biol. Rev.* **86**, e0004122 (2022). 35852448 10.1128/mmbr.00041-22 PMC9769567

[31] Borneman, W. S., Hartley, R. D., Morrison, W. H., Akin, D. E. & Ljungdahl, L. G. Feruloyl and *p*-coumaroyl esterase from anaerobic fungi in relation to plant cell wall degradation. *Appl. Microbiol. Biotechnol.* **33**, 345–351 (1990).

[32] Akin, D. E. & Benner, R. Degradation of polysaccharides and lignin by ruminal bacteria and fungi. *Appl Environ. Microbiol* **54**, 1117–1125 (1988). 3389808 10.1128/aem.54.5.1117-1125.1988 PMC202614

[33] Kajikawa, H. et al. Degradation of benzyl ether bonds of lignin by ruminal microbes. *FEMS Microbiol. Lett.* **187**, 15–20 (2000). 10828393 10.1111/j.1574-6968.2000.tb09129.x

[34] Joblin, K. & Naylor, G. E. Fermentation of woods by rumen anaerobic fungi. *FEMS Microbiol. Lett.* **65**, 119–122 (1989).

[35] Akin, D. E., Borneman, W. S. & Lyon, C. E. Degradation of leaf blades and stems by monocentric and polycentric isolates of ruminal fungi. *Anim. Feed Sci. Technol.* **31**, 205–221 (1990).

[36] Kondo, T., Ohshita, T. & Kyuma, T. Structural modifications of timothy lignin by *in vitro* rumen fermentation. *Ann. Zootech.* **44**, 153 (1995).

[37] Kondo, T., Ohshita, T. & Kyuma, T. Structural changes of forage grass lignin by rumen digestion: characteristics of soluble lignin released from timothy (*Phleum pratense* L.) by in vitro rumen digestion. *Jpn Agric. Res. Q.* **31**, 49–53 (1997).

[38] Susmel, P., Stefanon, B., Mills, C. R. & Spanghero, M. Rumen degradability of organic matter, nitrogen and fibre fractions in forages. *Anim. Prod.* **51**, 515–526 (1990).

[39] Susmel, P. & Stefanon, B. Aspects of lignin degradation by rumen microorganisms. *J. Biotechnol.* **30**, 141–148 (1993).

[40] Akin, D. E., Lyon, C. E., Windham, W. R. & Rigsby, L. L. Physical degradation of lignified stem tissues by ruminal fungi. *Appl. Environ. Microbiol.* **55**, 611–616 (1989). 16347869 10.1128/aem.55.3.611-616.1989 PMC184168

[41] Qi, M. et al. Isolation and characterization of a ferulic acid esterase (Fae1A) from the rumen fungus *Anaeromyces mucronatus*. *J. Appl. Microbiol.* **110**, 1341–1350 (2011). 21362116 10.1111/j.1365-2672.2011.04990.x

[42] Kim, H. & Ralph, J. Solution-state 2D NMR of ball-milled plant cell wall gels in DMSO-d_6_/pyridine-d_5_. *Org. Biomol. Chem.* **8**, 576–591 (2010). 20090974 10.1039/b916070a PMC4070321

[43] Mansfield, S. D., Kim, H., Lu, F. & Ralph, J. Whole plant cell wall characterization using solution-state 2D NMR. *Nat. Protoc.* **7**, 1579–1589 (2012). 22864199 10.1038/nprot.2012.064

[44] Dinh, C. V. & Prather, K. L. J. Development of an autonomous and bifunctional quorum-sensing circuit for metabolic flux control in engineered *Escherichia coli*. *Proc. Natl Acad. Sci. USA* **116**, 25562–25568 (2019). 31796590 10.1073/pnas.1911144116 PMC6926038

[45] Lu, F. & Ralph, J. Non-degradative dissolution and acetylation of ball-milled plant cell walls: high-resolution solution-state NMR. *Plant J.* **35**, 535–544 (2003). 12904215 10.1046/j.1365-313x.2003.01817.x

[46] Yelle, D. J. et al. A highly diastereoselective oxidant contributes to ligninolysis by the white rot basidiomycete *Ceriporiopsis subvermispora*. *Appl. Environ. Microbiol.* **80**, 7536–7544 (2014). 25261514 10.1128/AEM.02111-14 PMC4249248

[47] Yelle, D. J., Wei, D., Ralph, J. & Hammel, K. E. Multidimensional NMR analysis reveals truncated lignin structures in wood decayed by the brown rot basidiomycete *Postia placenta*. *Environ. Microbiol.* **13**, 1091–1100 (2011). 21261800 10.1111/j.1462-2920.2010.02417.x

[48] Oates, N. C. et al. A multi-omics approach to lignocellulolytic enzyme discovery reveals a new ligninase activity from *Parascedosporium putredinis* NO1. *Proc. Natl Acad. Sci. USA* **118**, e2008888118 (2021). 33903229 10.1073/pnas.2008888118 PMC8106297

[49] Dumond, L. et al. Termite gut microbiota contribution to wheat straw delignification in anaerobic bioreactors. *ACS Sustain. Chem. Eng.* **9**, 2191–2202 (2021).

[50] Love, M. I., Huber, W. & Anders, S. Moderated estimation of fold change and dispersion for RNA-seq data with DESeq2. *Genome Biol.* **15**, 550 (2014). 25516281 10.1186/s13059-014-0550-8 PMC4302049

[51] Kanehisa, M., Furumichi, M., Tanabe, M., Sato, Y. & Morishima, K. KEGG: new perspectives on genomes, pathways, diseases and drugs. *Nucleic Acids Res.* **45**, D353–D361 (2017). 27899662 10.1093/nar/gkw1092 PMC5210567

[52] Stecher, G., Tamura, K. & Kumar, S. Molecular Evolutionary Genetics Analysis (MEGA) for macOS. *Mol. Biol. Evol.* **37**, 1237–1239 (2020). 31904846 10.1093/molbev/msz312 PMC7086165

[53] Zhang, H. et al. DbCAN2: a meta server for automated carbohydrate-active enzyme annotation. *Nucleic Acids Res.* **46**, W95–W101 (2018). 29771380 10.1093/nar/gky418 PMC6031026

[54] Baek, M. et al. Accurate prediction of protein structures and interactions using a three-track neural network. *Science* **373**, 871–876 (2021). 34282049 10.1126/science.abj8754 PMC7612213

[55] Henske, J. K. et al. Metabolic characterization of anaerobic fungi provides a path forward for bioprocessing of crude lignocellulose. *Biotechnol. Bioeng.* **115**, 874–884 (2018). 29240224 10.1002/bit.26515

[56] Kim, K. H. & Kim, C. S. Recent efforts to prevent undesirable reactions from fractionation to depolymerization of lignin: toward maximizing the value from lignin. *Front. Energy Res.* **6**, 92 (2018).

[57] Solomon, K. V. et al. Catabolic repression in early-diverging anaerobic fungi is partially mediated by natural antisense transcripts. *Fungal Genet. Biol.* **121**, 1–9 (2018). 30223087 10.1016/j.fgb.2018.09.004

[58] Henske, J. K., Gilmore, S. P., Haitjema, C. H., Solomon, K. V. & O’Malley, M. A. Biomass-degrading enzymes are catabolite repressed in anaerobic gut fungi. *AIChE J.* **64**, 4263–4270 (2018).

[59] Hofrichter, M. Review: lignin conversion by manganese peroxidase (MnP). *Enzym. Microb. Technol.* **30**, 454–466 (2002).

[60] Orpin, C. G. & Greenwood, Y. Nutritional and germination requirements of the rumen chytridiomycete *Neocallimastix patriciarum*. *Trans. Br. Mycol. Soc.* **86**, 103–109 (1986).

[61] Theodorou, M. K., Davies, D. R., Nielsen, B. B., Lawrence, M. I. G. & Trinci, A. P. J. Determination of growth of anaerobic fungi on soluble and cellulosic substrates using a pressure transducer. *Microbiology* **141**, 671–678 (1995).

[62] Haitjema, C. H., Solomon, K. V., Henske, J. K., Theodorou, M. K. & O’Malley, M. A. Anaerobic gut fungi: advances in isolation, culture, and cellulolytic enzyme discovery for biofuel production. *Biotechnol. Bioeng.* **111**, 1471–1482 (2014). 24788404 10.1002/bit.25264

[63] Sluiter, A. *et al*. *Determination of Structural Carbohydrates and Lignin in Biomass: Laboratory Analytical Procedure (LAP).* Technical Report NREL/TP-510-42618 (National Renewable Energy Laboratory, 2012).

[64] Eudes, A. et al. Production of hydroxycinnamoyl anthranilates from glucose in *Escherichia coli*. *Microb. Cell Fact.* **12**, 62 (2013). 23806124 10.1186/1475-2859-12-62 PMC3716870

[65] Brown, J. L. et al. Co‑cultivation of the anaerobic fungus *Caecomyces churrovis* with *Methanobacterium bryantii* enhances transcription of carbohydrate binding modules, dockerins, and pyruvate formate lyases on specific substrates. *Biotechnol. Biofuels* **14**, 234 (2021). 34893091 10.1186/s13068-021-02083-w PMC8665504

[66] Henske, J. K. et al. Transcriptomic characterization of *Caecomyces churrovis*: a novel, non-rhizoid-forming lignocellulolytic anaerobic fungus. *Biotechnol. Biofuels* **10**, 305 (2017). 29270219 10.1186/s13068-017-0997-4 PMC5737911

[67] Peng, X., Gilmore, S. P. & O’Malley, M. A. Microbial communities for bioprocessing: lessons learned from nature. *Curr. Opin. Chem. Eng.* **14**, 103–109 (2016).

[68] Tuckwell, D. S., Nicholson, M. J., McSweeney, C. S., Theodorou, M. K. & Brookman, J. L. The rapid assignment of ruminal fungi to presumptive genera using ITS1 and ITS2 RNA secondary structures to produce group-specific fingerprints. *Microbiology* **151**, 1557–1567 (2005). 15870465 10.1099/mic.0.27689-0

[69] Wang, Q., Garrity, G. M., Tiedje, J. M. & Cole, J. R. Naïve Bayesian classifier for rapid assignment of rRNA sequences into the new bacterial taxonomy. *Appl. Environ. Microbiol.* **73**, 5261–5267 (2007). 17586664 10.1128/AEM.00062-07 PMC1950982

[70] Cole, J. R. et al. The Ribosomal Database Project: improved alignments and new tools for rRNA analysis. *Nucleic Acids Res.* **37**, D141–D145 (2009). 19004872 10.1093/nar/gkn879 PMC2686447

[71] Nilsson, R. H. et al. The UNITE database for molecular identification of fungi: handling dark taxa and parallel taxonomic classifications. *Nucleic Acids Res.* **47**, D259–D264 (2019). 30371820 10.1093/nar/gky1022 PMC6324048

[72] Lan, W. et al. Elucidating tricin–lignin structures: assigning correlations in HSQC spectra of monocot lignins. *Polymers* **10**, 916 (2018). 30960841 10.3390/polym10080916 PMC6403598

[73] Del Río, J. C. et al. Structural characterization of wheat straw lignin as revealed by analytical pyrolysis, 2D-NMR, and reductive cleavage methods. *J. Agric. Food Chem.* **60**, 5922–5935 (2012). 22607527 10.1021/jf301002n

[74] Guerra, A., Lucia, L. A. & Argyropoulos, D. S. Isolation and characterization of lignins from *Eucalyptus grandis* Hill ex Maiden and *Eucalyptus globulus* Labill. by enzymatic mild acidolysis (EMAL). *Holzforschung* **62**, 24–30 (2008).

[75] Bushnell, B. *BBMap: A Fast, Accurate, Splice-Aware Aligner* Report Number LBNL-7065E (Lawrence Berkeley National Laboratory, 2014).

[76] Kim, D., Langmead, B. & Salzberg, S. L. HISAT: a fast spliced aligner with low memory requirements. *Nat. Methods* **12**, 357–360 (2015). 25751142 10.1038/nmeth.3317 PMC4655817

[77] Kim, D., Paggi, J. M., Park, C., Bennett, C. & Salzberg, S. L. Graph-based genome alignment and genotyping with HISAT2 and HISAT-genotype. *Nat. Biotechnol.* **37**, 907–915 (2019). 31375807 10.1038/s41587-019-0201-4 PMC7605509

[78] Ramírez, F., Dündar, F., Diehl, S., Grüning, B. A. & Manke, T. deepTools: a flexible platform for exploring deep-sequencing data. *Nucleic Acids Res.* **42**, W187–W191 (2014). 24799436 10.1093/nar/gku365 PMC4086134

[79] Liao, Y., Smyth, G. K. & Shi, W. featureCounts: an efficient general purpose program for assigning sequence reads to genomic features. *Bioinformatics* **30**, 923–930 (2014). 24227677 10.1093/bioinformatics/btt656

[80] Van Rossum, G. & Drake, F. L. Jr *Python 3 Reference Manual*. (CreateSpace, 2009).

[81] Cock, P. J. A. et al. Biopython: freely available Python tools for computational molecular biology and bioinformatics. *Bioinformatics* **25**, 1422–1423 (2009). 19304878 10.1093/bioinformatics/btp163 PMC2682512

[82] Oliphant, T. E. *A Guide to NumPy* (Trelgol Publishing, 2006).

[83] Almagro Armenteros, J. J. et al. SignalP 5.0 improves signal peptide predictions using deep neural networks. *Nat. Biotechnol.* **37**, 420–423 (2019). 30778233 10.1038/s41587-019-0036-z

[84] Tsirigos, K. D., Peters, C., Shu, N., Käll, L. & Elofsson, A. The TOPCONS web server for consensus prediction of membrane protein topology and signal peptides. *Nucleic Acids Res.* **43**, W401–W407 (2015). 25969446 10.1093/nar/gkv485 PMC4489233

[85] Hiranuma, N. et al. Improved protein structure refinement guided by deep learning based accuracy estimation. *Nat. Commun.* **12**, 1340 (2021). 33637700 10.1038/s41467-021-21511-x PMC7910447

[86] Saitou, N. & Nei, M. The neighbor-joining method: a new method for reconstructing phylogenetic trees. *Mol. Biol. Evol.* **4**, 406–425 (1987). 3447015 10.1093/oxfordjournals.molbev.a040454

[87] Felsenstein, J. Confidence limits on phylogenies: an approach using the bootstrap. *Evolution* **39**, 783–791 (1985). 28561359 10.1111/j.1558-5646.1985.tb00420.x

[88] Jones, D. T., Taylor, W. R. & Thornton, J. M. The rapid generation of mutation data matrices from protein sequences. *Comput. Appl. Biosci.* **8**, 275–282 (1992). 1633570 10.1093/bioinformatics/8.3.275

[89] Kumar, S., Stecher, G., Li, M., Knyaz, C. & Tamura, K. MEGA X: Molecular Evolutionary Genetics Analysis across computing platforms. *Mol. Biol. Evol.* **35**, 1547–1549 (2018). 29722887 10.1093/molbev/msy096 PMC5967553

