# Supplementary material for: Lignin deconstruction by anaerobic fungi
Source: Nat Microbiol. 2023 Mar 9;8(4):596–610. doi: 10.1038/s41564-023-01336-8 (PMC10066034; doi:10.1038/s41564-023-01336-8)
Supplement: Supplementary file 1 — Supplementary text describing isolation of the Piromyces sp. E1M strain and activity assays using model compounds. [file 41564_2023_1336_MOESM1_ESM.pdf]

nature microbiology

Article
https://doi.org/10.1038/s41564-023-01336-8

Lignin deconstruction by anaerobic fungi

In the format provided by the authors and unedited

Supplementary text

Description and classification of the *Piromyces* sp. E1M strain

Preliminary characterization of the novel anaerobic fungal isolate from identified morphological characteristics are consistent with the genus *Piromyces* or *Neocallimastix*[9]. Light microscopy images of *Piromyces* sp. E1M suggest the isolate is monocentric with extensive microrhizoids and club shaped sporangia (Extended Data Fig. 2). Zoospores are motile, uniflagellate and ~5 µm in diameter, whereas sporangia appear ~20-40 µm across (Extended Data Fig. 2).

Amplification of marker genes and subsequent multiple sequence alignments support classifying this novel isolate as a *Piromyces*[69,70]. Multiple sequence alignment of the ITS1 gene, completed using the Ribosomal Database Project classifier tool, indicate a 98% probability that this sequence belongs to a fungus from the genus *Piromyces*. The LSU gene, classified by the same means, calculates a 100% probability that this sequence belongs to a *Piromyces*. The amplified ITS and LSU sequences have been deposited in GenBank under BioProject accession number PRJNA800048.

Model compound assays for interrogation of anaerobic lignin deconstruction mechanism

We used model compounds analogous to β-aryl-ether bonds in an attempt to reproduce the observed anti-lignin-bond activity *in vitro*[48]. Fractionating fungal cultures and assaying fraction's activities against the model demonstrated possible anti-β-O-4 activity in the small molecule (<10 kDa) fraction of fungal supernatants (Extended Data Fig. 7). We did not detect any cleavage of model compounds in the buffer-exchanged supernatant or in buffer-exchanged cell lysates which contained fungal proteins >10 kDa. We observed similar signal magnitudes and localizations within culture fractions for both *N. californiae* and *A. robustus* cultures. There are no proteins less than 10 kDa in the genes of interest we identified using RNA Seq.

The results of the activity assays against lignin model compounds suggest that the mechanisms by which anaerobic fungi facilitate lignin deconstruction is sensitive to environmental redox potential, and might be non-enzymatic. The lack of direct enzymatic activity against the model compound highlights the challenges associated with describing a novel biological process with no precedent, especially a process as complicated as biological lignin deconstruction. Aerobic biological lignin deconstruction is both redox sensitive and non-enzymatic, but is well established to occur. Despite establishing a working, aerobic redox enzyme as a positive control we ultimately could not observe anti-probe activity from the protein containing fractions we tested.

These results combined with observations of activity against diverse bond types in 2D-HSQC-NMR spectra lead us to hypothesize that the lignin remodeling we observe is non-enzymatic and mediated by small molecules, as opposed to direct enzyme-substrate interactions. In model compound assays, neither of the buffer-exchanged protein fractions (>10 kDa) exhibited activity, but the filtrate of the supernatant fraction did and only when oxygen was added. This might suggest that a fungal enzyme or enzyme suite is facilitating lignin remodeling through indirect, small molecule-mediated redox mechanisms, possibly analogous to reactions facilitated by oxidases of higher fungi[6,7]. Alternatively, the activity we observe could be analogous to how these enzymes function *in situ* since rumination serves to oxygenate rumen contents. Regardless of the source, anaerobic fungi are equipped to encounter reactive oxygen species, as demonstrated by their suite of encoded oxygen tolerance enzymes. The localization of

the anti-β-O-4 signal to the supernatant, as opposed to an intracellular localization, is logical since the size of lignin oligomers likely precludes their intact transport across cell walls and fungal membranes.
