# Supplementary material for: Lignin deconstruction by anaerobic fungi
Source: Nat Microbiol. 2023 Mar 9;8(4):596–610. doi: 10.1038/s41564-023-01336-8 (PMC10066034; doi:10.1038/s41564-023-01336-8)
Supplement: Supplementary file 2 — Reporting Summary [file 41564_2023_1336_MOESM2_ESM.pdf]

nature portfolio

Corresponding author(s): Prof. Michelle A. O'Malley
Dr. Thomas S. Lankiewicz

Last updated by author(s): Dec 16, 2022

Reporting Summary

Nature Portfolio wishes to improve the reproducibility of the work that we publish. This form provides structure for consistency and transparency in reporting. For further information on Nature Portfolio policies, see our Editorial Policies and the Editorial Policy Checklist.

Statistics

For all statistical analyses, confirm that the following items are present in the figure legend, table legend, main text, or Methods section.

n/a	Confirmed	
☐	☒	The exact sample size (*n*) for each experimental group/condition, given as a discrete number and unit of measurement
☐	☒	A statement on whether measurements were taken from distinct samples or whether the same sample was measured repeatedly
☐	☒	The statistical test(s) used AND whether they are one- or two-sided<br>*Only common tests should be described solely by name; describe more complex techniques in the Methods section.*
☐	☒	A description of all covariates tested
☐	☒	A description of any assumptions or corrections, such as tests of normality and adjustment for multiple comparisons
☐	☒	A full description of the statistical parameters including central tendency (e.g. means) or other basic estimates (e.g. regression coefficient) AND variation (e.g. standard deviation) or associated estimates of uncertainty (e.g. confidence intervals)
☐	☒	For null hypothesis testing, the test statistic (e.g. *F*, *t*, *r*) with confidence intervals, effect sizes, degrees of freedom and *P* value noted<br>*Give P values as exact values whenever suitable.*
☐	☒	For Bayesian analysis, information on the choice of priors and Markov chain Monte Carlo settings
☐	☒	For hierarchical and complex designs, identification of the appropriate level for tests and full reporting of outcomes
☐	☒	Estimates of effect sizes (e.g. Cohen's *d*, Pearson's *r*), indicating how they were calculated

Our web collection on statistics for biologists contains articles on many of the points above.

Software and code

Policy information about availability of computer code

Data collection	Agilent's OpenLab CDS (version 2.6) and MassHunter (version 11.0) were used to collect HPLC/GPC and LC-MS data, respectively. Bruker's TopSpin software (version 4.1.0) was used to aquire 2D-HSQC-NMR data. Tecan's i-control software (verison 2.0) was used to acquire plate reader data.
Data analysis	The HPLC data were integrated in OpenLabCDS (version 2.6) and LC-MS data were integrated in MassHunter (version 11.0). The 2D-HSQC-NMR data were analyzed in MestreNOVA (version 14). Differential expression data were quality controlled and aligned using BBDuk (version 38.90), HISAT2 (version 2.1.0), deeptools (version 3.1), FeatureCounts (version 2.0.0), and statistically analyzed using DESeq2 (version 1.28.1). All other statistical analyses were conducted in GraphPad Prism (version 9.3.1). Various predictive models such as dbCAN (version 3.0.1), SignalP (version 5.0), TOPCONS (version 2.0), and RoseTTAfold were used to predict features of genes that were differentially expressed. Differential expression data and associated predictions were parsed using Python (version 3.9.13), bioPython (version 1.79), and NumPy (version 1.23.1).

For manuscripts utilizing custom algorithms or software that are central to the research but not yet described in published literature, software must be made available to editors and reviewers. We strongly encourage code deposition in a community repository (e.g. GitHub). See the Nature Portfolio guidelines for submitting code & software for further information.

Data

Policy information about availability of data

All manuscripts must include a data availability statement. This statement should provide the following information, where applicable:

- Accession codes, unique identifiers, or web links for publicly available datasets
- A description of any restrictions on data availability
- For clinical datasets or third party data, please ensure that the statement adheres to our policy

All data are available in the main text, the Extended Data, the Supplementary Information, or through JGI's Mycocosm (https://mycocosm.jgi.doe.gov/mycocosm/home). Raw reads of differential expression data are deposited in NCBI's Sequence Read Archive database and can be found with SRA accession numbers SRP288871-SRP288885. Amplicon sequences for fungal taxonomic classification are deposited in GenBank under BioProject accession number PRJNA800048. The Cazyme database (http://www.cazy.org/), used for active site predictions, and the AlphaFold protein database (https://alphafold.ebi.ac.uk/), used for protein structural predications, are both publicly available.

Field-specific reporting

Please select the one below that is the best fit for your research. If you are not sure, read the appropriate sections before making your selection.

☒ Life sciences ☐ Behavioural & social sciences ☐ Ecological, evolutionary & environmental sciences

For a reference copy of the document with all sections, see nature.com/documents/nr-reporting-summary-flat.pdf

Life sciences study design

All studies must disclose on these points even when the disclosure is negative.

Sample size	In all cases experiments were conducted using biological triplicates to support downstream statistical tests. In most cases no technical replication was performed due to the high precision of the methods used. In the case of florescence data, acquired on the Tecan M200 plate reader, reactions were sampled five times for technical replication and error from companion measurements were propagated to provide an appropriate estimation of error.
Data exclusions	There are no data excluded from our analyses.
Replication	All microbial cultivation experiments were done using biological triplicates in order to ensure that measurements were reproducible, and results could be statistically tested once acquired.
Randomization	Randomization was not relevant to this study as it compared microbial cultures in highly controlled experimental conditions in which all other conditions other than the experimental treatment were kept the same. For example, in the microbial cultivation experiments described in this manuscript covariates were controlled by using the same batches of media batch for various experimental conditions and the same inoculum for experimental comparisons.
Blinding	Blinding was not relevant to this study as it compared microbial cultures in highly controlled experimental conditions in which the difference in treatment were applied by those conducting the experiments and all other conditions other than the experimental treatment were kept the same.

Reporting for specific materials, systems and methods

We require information from authors about some types of materials, experimental systems and methods used in many studies. Here, indicate whether each material, system or method listed is relevant to your study. If you are not sure if a list item applies to your research, read the appropriate section before selecting a response.

Materials & experimental systems

n/a	Involved in the study	
☒	☐	Antibodies
☒	☐	Eukaryotic cell lines
☒	☐	Palaeontology and archaeology
☐	☒	Animals and other organisms
☒	☐	Human research participants
☒	☐	Clinical data
☒	☐	Dual use research of concern

Methods

n/a	Involved in the study	
☒	☐	ChIP-seq
☒	☐	Flow cytometry
☒	☐	MRI-based neuroimaging

Animals and other organisms

Policy information about studies involving animals; ARRIVE guidelines recommended for reporting animal research

Laboratory animals	This study did not involve laboratory animals.
Wild animals	This study did not involve wild animals.
Field-collected samples	There was no detailed protocol, or specified conditions, for collection animal fecal samples for this study. Feces were collected within several hours of donation under aerobic conditions, as detailed in our previous publications. These publications are cited in the relevant methods section.
Ethics oversight	No ethical approvals of these procedures were required since the gut microbiomes of domesticated animals were sampled noninvasively by collecting feces from animal pens.

Note that full information on the approval of the study protocol must also be provided in the manuscript.
